# New Peptaibiotics and a Cyclodepsipeptide from *Ijuhya vitellina*: Isolation, Identification, Cytotoxic and Nematicidal Activities

**DOI:** 10.3390/antibiotics9030132

**Published:** 2020-03-22

**Authors:** Ashaimaa Y. Moussa, Christopher Lambert, Theresia E.B. Stradal, Samad Ashrafi, Wolfgang Maier, Marc Stadler, Soleiman E. Helaly

**Affiliations:** 1 Department of Microbial Drugs, Helmholtz Centre for Infection Research, Inhoffenstraße 7, 38124 Braunschweig, Germany; ashaimaa_yehia@pharma.asu.edu.eg; 2 Department of Pharmacognosy, Faculty of Pharmacy, Ain shams University, Abbassia, 11566 Cairo, Egypt; 3 Department of Cell Biology, Helmholtz Centre for Infection Research (HZI), Inhoffenstraße 7, 38124 Braunschweig, Germany; Christopher.Lambert@helmholtz-hzi.de (C.L.); Theresia.Stradal@helmholtz-hzi.de (T.E.B.S.); 4 Institute for Epidemiology and Pathogen Diagnostics, Julius Kühn-Institut (JKI)–Federal Research Centre for Cultivated Plants, Messeweg 11/12, 38104 Braunschweig, Germany; samad.ashrafi@julius-kuehn.de (S.A.); wolfgang.maier@julius-kuehn.de (W.M.); 5 Department of Chemistry, Faculty of Science, Aswan University, 81528 Aswan, Egypt

**Keywords:** cyclodepsipeptides, nematicidal activity, peptaibiotics, Sordariomycetes, structure elucidation

## Abstract

Fungal associations with nematodes have attracted scientific attention because of the need to develop new biocontrol agents. In this context, *Ijuhya vitellina*, an antagonistic fungus previously isolated from the plant parasitic cyst nematode *Heterodera filipjevi*, was selected to carry out an in-depth metabolomic study for its active metabolites. Herein, three new nonapeptide peptaibols with leucinostatin based sequences were isolated and identified by 1, 2D NMR, and HR-ESI-MS-MS. The absolute configuration was assigned based on Marfay’s analysis and Mosher ester formation. The new leucinostatins manifested moderate nematicidal effect against the plant pathogenic nematode *Pratylenchus penetrans* with LD_90_ values ranging from 5 to 7 µg/mL. Furthermore, a cyclodepsipeptide, named arthrichitin D, with five amino acid residues attached to a 3-hydroxy-2,4-dimethylhexadeca-4,6-dienoic fatty acid chain was discovered and showed weak nematicidal effect against *Caenorhabditis elegans*. Chaetoglobosin B and its 19-*O*-acetyl derivative were also obtained as minor metabolites, and the activity of chaetoglobosin B on the actin cytoskeleton of mammalian cells was assessed.

## 1. Introduction 

Being able to infect animals, microorganisms, and plants, nematodes have been the subject of intense research in the last decade; particularly, plant nematodes that were reported to cause huge losses in the agricultural field among many of the economically influential crops with more than US 80$ billion estimated annual cost. Root-knot nematodes (*Meloidogyne* spp.), cyst nematodes (especially *Heterodera* and *Globodera* spp.), as well as root lesion nematodes (*Pratylenchus* spp.) are among the most destructive plant parasitic nematodes due to their complex biotrophic parasitism and multiple developmental stages [1,2,3].

Even though synthetic chemicals as methyl bromide were used efficiently to combat nematodal pathogens in soil, they turned out to be powerful toxins to all soil living organisms as well as demolishing agents to the ozone layer. Consequently, they were banned to avoid environmental and health disastrous effects. This raised the need for safe alternatives for pest control in both agriculture and forestry. Biological control featured a plausible and favorable solution where nematophagous fungi or their secondary metabolites were introduced to repel and destroy plant parasites [4,5]. Indeed, metabolic pathways of fungi were proven to present unprecedented carbon skeletons whose biological effects could offer superior prospects; for example, omphalotin A from the basidiomycete *Omphalotus olearius* represented a lead compound for development of an agrochemical nematicide against *Meloidogyne incognita* [6,7], but was finally abandoned because of unfavorable costs of goods.

A recent survey of the cyst nematode *Heterodera filipjevi* collected from wheat fields in Turkey and the establishment of a novel isolation technique resulted in the discovery of several new genera and species of nematode-antagonistic fungi [8,9]. These recently described fungal antagonists of nematodes are potential candidates for biocontrol of nematodes and are also currently being investigated for their secondary metabolite production because any biocontrol agent must be proven to be safe and should, e.g., not overproduce mycotoxins [10,11]. *Ijuhya vitellina* (ex-type strain DSM 1004495), one of the aformementioned antagonists, was recently discovered and found to produce chaetoglobosin A and its 19-*O*-acetyl derivative as major secondary metabolites [10]. Since extracts of this fungus showed interesting metabolite profiles during the course of a variation of culture media, we have continued to characterize its secondary metabolites and here describe the isolation, structural elucidation and biological characterization of the obtained molecules. These data will be valuable to assess the potential of *I vitellina* for its use as biocontrol agent against plant parasitic nematodes.

## 2. Results and Discussion

### 2.1. Isolation and Structure Elucidation of Secondary Metabolites

As described in the experimental part, *I. vitellina* was cultured in liquid Q6 medium for 12 days until glucose depletion. This coincided with the optimum of secondary metabolite production. Extraction of the mycelia as described in the Experimental yielded 900 mg of material, from which four previously undescribed metabolites were obtained and identified by spectral analysis. For these oligopeptides, we propose the trivial names leucinostatins U, P, Q, and arthrichitin D and describe their physico-chemical characteristics below. In addition, two known chaetoglobosins, chaetoglobosin B, and 19-*O*-acetyl-chaetoglobosin B were isolated and identified from the same extract.

**Figure 1:**
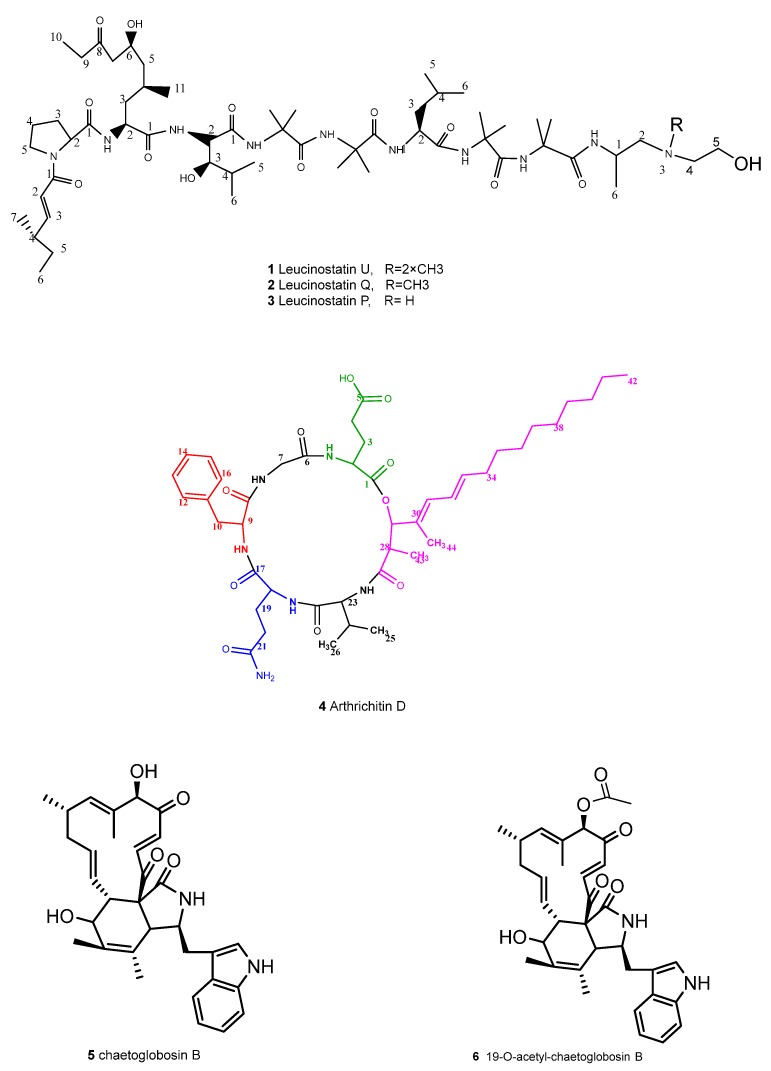
Chemical structures of the metabolites isolated from *Ijuhya vitellina* DSM 104495.

**Figure 2:**
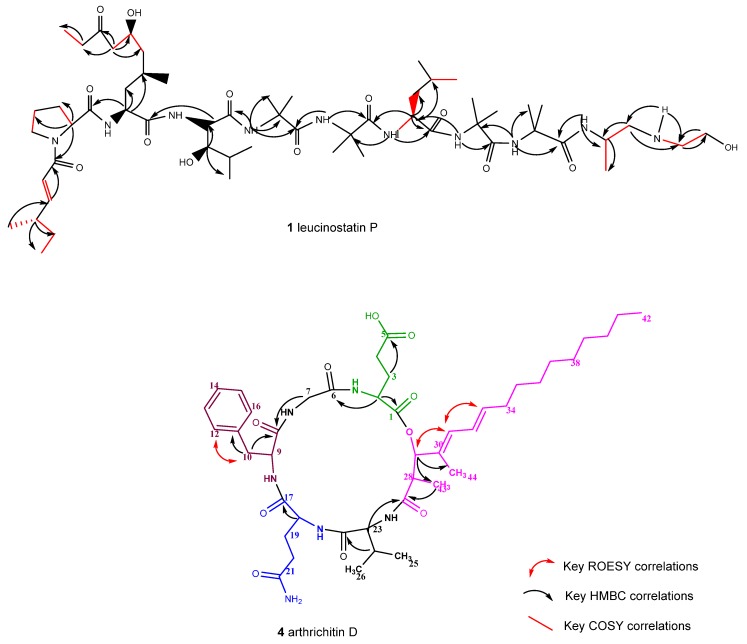
2D NMR correlations of compounds **1** and **4** from *Ijuhya vitellina*.

Leucinostatin U (**1**) was isolated as amorphous orange yellow powder. The HR-ESI-MS spectrum indicated a molecular formula of C_58_H_105_N_10_O_13_ corresponding to a mass of 1149.7864 [M + H]^+^ and 12 degrees of unsaturation. As compared to leucinostatin Y [12], it lacked two carbons, one hydrogen, and two oxygens, which was confirmed by ^13^C-NMR and ^1^H-NMR spectroscopy. The complete assignments of all carbons are listed in Figure 1, Figure 2, and Figure S1 (available in supplementary data). According to the ^13^C-NMR and HSQC spectra, 58 carbons were detected and could be classified as 14 quaternary, 12 methylene, 19 methyls, 13 methines, and one quaternary substituted nitrogen. Moreover, 8 amide protons (N-1 to N-9) were recorded from the ^1^HNMR and the HSQC spectra. All the spin systems were proven to exist from the TOCSY correlations between the coupled protons in **1**. The long range correlations among protons and carbons were revealed from the HMBC spectra. All the data manifested the occurrence of a 4-methyl-2-hexenoic acid, proline (Pro), 6-hydroxy-4-methyl-8-oxodecanoic acid (AHMOD), β-hydroxy leucine (HyLeu), one leucine (Leu), and four 2-aminoisobutyric acid (Aib) residues and a C- terminal part comprised of a 2-dimethylamino-ethan-1-ol moiety (Figure 1 and Figure 2). Therefore, we deduced that **1** lacked one leucine, possessed one extra Aib residue and a C-terminal 2-dimethylamino-ethan-1-ol instead of the *β*-alanine and the alanine residues in leucinostatin Y.

By surveying the HMBC spectra, we could determine the order of attachment of the different residues due to the presence of correlations between the carbonyl of the 4-methyl, 2-hexenoic acid moiety, and the alpha proton of the proline amino acid (δH 4.20), the alpha proton (δH 4.09) of the AHMOD and the carbonyl of proline (δC 174.76), the alpha proton of HyLeu and the carbonyl of the AHMOD, the NH (δH 8.47) of Aib1 and the carbonyl (δC 172.48) of HyLeu, the NH (δH 7.56) of Aib2 and the carbonyl of the Aib1 (δC 174.50), the NH (δH 7.75) of Leu and the carbonyl (δC 176.03) of Aib2, the NH (δH 8.12) of Aib3 and the carbonyl (δC 174.89) of Leu, the NH (δH 7.16) of Aib4 and the carbonyl (δC 173.90) of Aib3, the NH (δH 7.35) of the C-terminal part and the carbonyl (δC 173.94) of the Aib4.

High resolution ESI-MS-MS was utilized for determination of the fragmentation pattern (suppementary Figure S2) and the 2D structure of leucinostatin **U 1** (Figure 2). These fragments *m/z* 208.1331, 421.2708, 550.3487, 635.4015, 720.4539, 833.5377, 918.5907, 1003.6436, 1060.7023, as a sequence of nine amino acids (the N-terminal part) were detected and this was further confirmed by the COSY, HMBC and TOCSY correlations (see supplementary, S7, S8, S9).

The absolute configuration of alpha carbons of the assigned amino acids (proline and leucine) was determined by the advanced Marfay’s analysis. Leucinostatin **U** (**1**; 0.5 mg) was hydrolyzed in 6 M HCl at 120 °C for 24 h followed by addition of the l-form of 5-fluoro-2,4-dinitrophenylleucineamide (FDLA). Subsequently, the complexes formed with l-and d-proline and l- and d-leucine were detected by liquid chromatography-mass spectrometry (LC-MS) giving the actual masses of 368.15 and 384.18 *m/z*, respectively. The retention times of the two complexes were 5.4 min and 7.4 min, respectively, which confirmed the L-configuration of both the proline and leucine residues (supplementary Figure S3).

To disclose the 3D-configuration of secondary alcohols in the HyLeu and the AHMOD moieties, a Mosher ester reaction was carried out. Leucinostatin U **1** was dissolved in dry pyridine to realize the Mosher esters’ formation. The chloride derivatives of the α-methoxy-α-trifluoromethylphenylacetyl ((*R*)-(−)-MTPA-Cl and (*S*)-(+)-MTPA-Cl) were added, taking into consideration that the (*R*)-(−)-MTPA-Cl yields the (*S*)-MTPA ester and vice versa. Following esterification, the products were subjected to ^1^HNMR, HSQC and COSY to measure the modified chemical shifts and calculate the differences (Δδ SR = ΔS −ΔR) between the original ^1^HNMR and those of the MTPA esters (see supplementary Figure S4 and Table S5). For the AHMOD, C6 was regarded as 6S as indicated by the positive sign chemical shifts caused by the O-MTPA phenyl group with a *J*-value of 7 Hz (detected from the J-resolved analysis), yet C4 was completely overlapped and could not be examined even with the J-resolved analysis. As for the HyLeu, the chemical shift calculations from the Mosher esters (S-MPTA) and the (R-MPTA) revealed that C3 was 3R, and the alpha proton of the HyLeu assumed the R configuration as well (as indicated from the C3 *J*-value 2.5 Hz). As to the C-terminal 3D-configuration at C-1, it remained undetermined due to the insufficient material to do further analysis. As a result, the absolute configuration of the secondary alcohols in the AHMOD and HyLeu amino acids were assigned based on the sign obtained from the chemical shift difference. The S configuration of the AHMOD was in accordance with that of leucinostatin Y; additionally, the alpha proton of the HyLeu was described as l- and its secondary alcohol was determined by Mosher to be R. Circular dichroism correlations were used to further confirm the absolute configuration of **1**, as they were shown to be in close accordance with leucinostatin A with a negative maximum at 203 nm and a large shoulder at 210–220 nm; hence, the alpha proton of the AHMOD amino acid was considered as R configuration [13]; therefore, it can be inferred that its stereochemistry is l-proline, l-leucine, 3R, l-HyLeu, 2R, 6S-AHMOD.

Leucinostatin Q (**2**) was isolated as an amorphous yellow powder with a molecular formula of C_57_H_103_N_10_O_13_ and 12 degrees of unsaturation based on the HR-ESI-MS, *m/z* 1135.7701 [M + H]^+^. After analysis of the ^1^H and ^13^C-NMR, it was evident that **2** was another derivative of the leucinostatin class with high resemblance to **1** (Table 1). The molecular weight suggested the absence of one methyl group (14 Da) as compared to **1**. Close examination of the NMR data revealed a change in the C-terminal part with the absence of the characteristic signals at (C-2, δC 68.65), (C-3, δC 66.59), (C-4, δC 55.48), and the lack of two *N*-methyl protons forming the quaternary nitrogen at (C-5 and C-6, δC 51.91). In addition, the methyl group (C-7, δC 20.86) was still detected in **2** with an additional *N*-methyl signal at δC 43.41 ppm. Up fielded proton and carbon chemical shifts were recorded at C-1, to be at 3.81 instead of 4.40 ppm, C-2 and C-3 to be 2.31 (63.31, CH2) and 2.45(60.21, CH2), which supported the structure of the C-terminal fragment to be a methyl amino ethan-1-ol moiety. This hypothesis was further affirmed through the COSY, TOCSY and HMBC correlations (see supplementary, S12–S16).

Leucinostatin P (**3**) was purified as yellowish powder, and the molecular formula was assigned to be C_56_H_101_N_10_O_13_ according to the HR-ESI-MS analysis with *m/z* 1121.7544 [M + H]^+^. A comparison of the ^1^H and ^13^C-NMR data revealed that **3** had identical chemical shifts to **2** except for the upfield shift of C-2 and C-3 in the C-terminal part (δC 54.13 and δC 51.12 vs δC 63.31 and δC 60.21, respectively), owing to the absence of the N-methyl group. Additionally, the NOESY correlations supported the above-mentioned derivation (see supplementary data S20–S24). The three compounds leucinostatins U, Q and P only differ in their degree of N-methylation in the C-terminal part.

Arthrichitin D (**4**) was obtained as pale yellow powder with a molecular formula of C_44_H_66_N_6_O_10_ deduced from the HR-ESI-MS *m/z* 839.4913 [14]. The well resolved ^1^H-NMR and ^13^C-NMR spectra (shown in Figure 1 and Figure 2, Supplementary S25), and the five exchangeable protons at (δC 7.35, δC8.06, δC 8.81, δC 7.97, δC 7.98) in DMSO-*d*_6_ confirmed the structure. Close examination of the NMR data showed the presence of phenylalanine (δC 126.28, 129.5 &130.5), valine (δC 55.6, 33.27, 19.17, 17.7), glycine (δC 43.4, δH 3.73, and 3.90), glutamate (δC 173.02, 169.92, glutamine (δC 171.60, 170.16) amino acid moieties and a long chain of unsaturated hydrocarbons. HMBC and COSY correlations demonstrated the spin systems of the five amino acids and confirmed their attachment. Thus, **4** was established as a new arthrichitin derivative, named arthrichitin D. The relative configuration of the 2,4-dimethylhexadeca-4,6-dienoic fatty acid chain was determined from the ^1^H, ^1^H ROESY correlations where a clear signal was detected between H-29, H-32, and H-31 indicating the *E*-configuration of Δ30, which is further confirmed by the upfield ^13^C-NMR shift of the methyl C-44 (δC 11.24) as it is trans to an alkyl group rather than a proton. The double bond Δ32 was proved to have *E*-configuration as well, due to the vicinal coupling constant of 15.2 Hz between H-33 and H-32 and further corroborated by the absence of any ROESY signals between H-31 and H-34. (Supplementary S27–S31)

Among the linear peptides named peptaibols or peptaibiotics, leucinostatins are lipoaminopeptides that were first isolated from cultures of the fungus *Purpureocillium lilacinum* (previously named *Paecilomyces lilacinus*). Peptaibols are characterized by an amino acid sequence between 5 and 21 units, N-terminal acylated residues and a number of amino-isobutyric (Aib) modified amino acids with a C-terminus in the form of amide-bonded or acetylated 1,2-amino alcohol. While leucinostatin Y showed the ability to inhibit mitochondrial ATPase in PANC-1 cells, its analog, leucinostatin A, revealed immunosuppressant effect through acting as a weak ionophore in T-lymphocytes. Leucinostatins were the most potent amidst other screened peptides against trypanosomes [15,16,17,18,19].

From a peptaibiotic repository of more than 1297 sequences, twenty six leucinostatins were discovered until today, yet only few pure compounds were assayed against nematodes. These peptides were first referred to as nematicides in 2004 when a mixture of leucinostatins B, D, F, H, L, and T was reported to kill a combination of juvenile and adults of a *C. elegans* population with 77% and 100% mortality rates after 2 and 12 h, respectively, at an overall concentration of 100 μg/mL and 74% mortality after 24 h at the concentration of 10 μg/mL using 2% methanol as a negative control. While these lipopeptides and other chemically diverse molecules from nematode-associated fungi may feature a broad spectrum of activity, they are a great foundation for developing future therapeutics with advanced selectivity [6,18,20].

### 2.2. Nematicidal Activity of the Metabolites of I. vitellina

The microtiter plate assay for nematicidal activity (Table 1) revealed that **1** possessed weak nematicidal effects against *Pratylenchus penetrans*, yet it showed stronger activity against *C. elegans*, a free-living nematode that is generally more sensitive than the plant parasites. Compounds **1**–**3** showed moderate nematicidal activity against *C. elegans*, but they were inactive against *P. penetrans* (Table 1). Arthrichitin D **4** was inactive against both, *C. elegans* and *P. penetrans*, while the chaetoglobosin derivative **6** showed moderate to weak activity against *C. elegans* only. Compound **5** was not tested because only a small amount of pure material was available and this was used for the evaluation of actin inhibition.

### 2.3. Assessment of Chaetoglobosin B Activity on Actin

The influence of chaetoglobosin B on the actin cytoskeleton of the mammalian cell line U2OS was analyzed by fluorescence microscopy using a fluorescently labelled phalloidin to allow tracking of changes in filamentous actin (F-actin) formation and 4’,6-diamidino-2-phenylindole (DAPI) to visualize nuclei. Increasing concentrations of chaetoglobosin B from 0.01 to 1 µg/mL were used to survey in detail observation of toxicity since comparable effects have been observed for chaetoglobosins A and –D [21]. With rising concentration, F-actin starts to aggregate in the cytoplasm visible as knot-like structures (**A**–**C**), followed by deterioration of the cell periphery comparable to the effect of other cytochalasans (**D**–**E**) [21,22]. It will be very interesting to perform structure–function analyses and better understand the molecular mechanism of chaetoglobosins on F-actin in animal cells, also given the increasing number of chaetoglobosins that are being continuously discovered [23,24].

### 2.4. Conclusion

The current study revealed that the potential biocontrol candidate *I. vitellina* is able to produce a substantial diversity of secondary metabolites, which comprise oligopeptides as well as cytochalasins of the chaetoglobosin type. The production of these compounds is dependent on the culture conditions. From the results of our preliminary biological characterization, it can be assumed that the newly discovered oligopeptides as well as the chaetoglobosins contribute to the antagonistic potential of the fungus. These metabolites should in the future be made available in larger quantities and be checked carefully in a more detailed evaluation including cytotoxic and antimicrobial effects. Presently, the producer strain is being subjected to a scale-up of fermentation to stirring bioreactors. The material derived from such large scale production campaigns can be used for preparative isolation of the target molecules, and it is planned to conduct more bioassays with the pure metabolites once they will become available in sufficient quantities. It will be interesting in particular to further study the effects of the chaetoglobosins on actin and mammalian cells, using state of the art methodology. At the same time, it remains to be seen whether the new fungus can be grown at large scale in an economical manner to provide enough inoculum for greenhouse and—later on—field studies, including development of suitable formulations. Despite the fact that many obstacles will have to be overcome until a commercial biocontrol agent can be developed, the results appear very promising.

## 3. Materials and Methods

### 3.1. General Information

UV spectra were recorded using methanol with a UV-vis spectrophotometer UV-2450, Shimadzu (Duisburg, Germany); optical rotations were measured with a Perkin-Elmer (Washington, USA) 241 MC spectrometer by employing a 1 mL volume quartz cuvette with 10 cm path length and sodium D line. A JASCO (Darmstadt, Germany) spectropolarimeter type J-815 was used to record the CD spectra, and compounds were dissolved in 500 µL in a 1 mm quartz cuvette. NMR data were recorded with a Bruker AVII-600 spectrometer equipped with a BBFO SmartProbe, ^1^H 500 MHz and ^13^C 150 MHz, as well as a Bruker Ascend III spectrometer, ^1^H 700 MHz and ^13^C 175 MHz, (Bruker Daltonics, Bremen, Germany). HPLC/DAD/MS were performed on an amaZon Speed ETD ion trap mass spectrometer (Bruker Daltonics), and HR-ESI-MS spectra were conducted on a maXis ESI-TOF (Bruker Daltonics) mass spectrometer, system; column 2.1 × 50 mm, 1.7 m, UPLC, C18 (Waters GmbH, Eschborn, Germany), solvent A: water + 0.1% formic acid, solvent B: acetonitrile + 0.1% formic acid, gradient: 5% B for 0.5 min increasing to 100% B in 19.5 min and then maintaining 100% B for 5 min, UV/Vis spectra was detected in the range of 200–600 nm combined with ESI-TOF-MS with a flow rate of 0.6 mL/min. (Scan range 100–2500 *m/z*, temperature 200 °C, capillary voltage 4500 V). The isolation of bioactive metabolites was carried out by employing an Agilent (Agilent, Waldbronn, Germany) 1100 series preparative HPLC system as described previously [11]. HPLC grade solvents were obtained from Merck Co. (Darmstadt, Germany), and chemicals were purchased from AppliChem GmbH (Darmstadt, Germany) and Carl Roth GmbH (Karlsruhe, Germany).

### 3.2. Fermentation and Extraction

*Ijuhya vitellina* strain DSM104495 was cultured on PDA (potato dextrose agar) plates for 3–4 weeks until the culture showed development of a characteristic orange pigmentation. Subsequently, small agar plugs were excised by a cork borer (5 mm diam.) to inoculate a 5 L fermentation batch culture composed of 25 sterilized 500 mL Erlenmeyer flasks. Each flask was filled with 200 mL of Q6 medium (d-glucose 2.5 g/L, glycerol 10 g/L, cotton seed flour 5 g/L [10]) and adjusted to pH 7.2 before autoclaving at 121 °C for 15 min. Until the complete consumption of glucose, cultures were incubated at 23 °C for 12 days in the dark and shaken at 140 rpm on a rotary shaker. Three days after the glucose was used up, HPLC analysis revealed that secondary metabolite production had stagnated. The mycelia were separated from the supernatant by vacuum filtration and extracted three times with acetone in an ultrasonic bath. Then, the mycelia were further processed to isolate the peptides (compounds **1**–**4**), while the culture filtrate was used to isolate the chaetoglobosins (**5**–**6**).

Following the evaporation of the combined mycelial acetone extract, the aqueous phase was extracted by an equal volume of ethyl acetate. While the aqueous layer was discarded, the organic phase was collected, dried over anhydrous sodium sulphate, and evaporated until dryness to yield 0.9 g of mycelial extract.

The adsorbent resin Amberlite XAD-16N (Sigma-Aldrich, Darmstadt, Germany) (50 g per 1 L) was used to extract the supernatant; subsequently, the XAD was extracted with acetone three times under ultrasonic conditions for 30 min at 40 °C followed by the same protocol mentioned above for the mycelial extract.

### 3.3. Isolation of ***1***–***6***

The total yellowish brown mycelial extract was filtered over a RP-C18 solid cartridge (Strata-X 55 mm, Phenomenex, Aschaffenburg, Germany) to give a 0.9 g of the crude extract.

The isolation of secondary metabolites was conducted using a preparative HPLC (Gilson, Middleton, USA) provided with GX-271 handler, a 305 pump (50SC pump piston head) and a photodiode array detector (DAD 210). The stationary phase used was a C18 Nucleodur 100-10 (Macherey-Nagel), column 150 × 40 mm, 7 μm. Mobile phase consisted of deionized water, prepared with a MilliQ (Millipore, Schwalbach, Germany) device, and 0.1% formic acid (solvent A) and acetonitrile AcCN + 0.1% formic acid (solvent B). The separation gradient was composed of: linear flow from 5% B for 5 min and increasing to 100% B in 60 min; subsequently, maintaining 100% B for 5 min at flow rate 35 mL/min to yield the major fractions. Further purification was led on an Agilent 1100 series preparative HPLC system (Agilent Technologies) using a Kromasil 100 C18 (20 × 250 mm, 7 µm,) as stationary phase. The mobile phase was composed of solvent A: H_2_O + 0.1% formic acid and solvent B: AcCN + 0.1% formic acid. The gradient started with isocratic conditions (5% B for 5 min), followed by a linear gradient increasing to 100% B in 40 min and then maintaining 100% B for 5 min with a flow rate of 20 mL/min. Fractionation was done according to UV peaks in the chromatograms recorded at 210, 280 and 354 nm. Compounds **1** (0.67 mg) and **2** (20 mg) were obtained in a pure form at t_R_ of 10.6 min and 10.4 min, respectively. Compound **3** (13.6 mg) eluted at 8-9 min. Compound **4** (0.7 mg) was obtained at 13–15 min.

Compounds **5** and **6** were purified at t_R_ 8.6 and 10.1 min, respectively, from the supernatant extract using a preparative HPLC (Gilson, Middleton, USA) under the same conditions as described above.

**Leucinostatin U (1):** 0.67 mg of yellow powder, ${\left[\alpha \right]}_{D}^{20}$ +30 (c 0.5, MeOH); UV (MeOH) λ_max_ (log ε) 221 nm, 192 nm. LCMS *m/z* 1149.7 [M + H]^+^ 575.3970(50) HR-ESI-MS *m/z* 1149.7864 [M + H]^+^ (calcd for C_58_H_10_5N_10_O_13_), LC-MS/MS data see supplementary information (S2). ^1^H-NMR and ^13^C-NMR spectroscopic data see supplementary information (S1).

**Leucinostatin Q (2):** 20 mg of orange powder, ${\left[\alpha \right]}_{D}^{20}$ +54 (c 0.5, MeOH); UV (MeOH) λ_max_ (log ε) 222 nm, 193 nm, 223 (sh 4.27). LCMS *m/z* 1135.7 [M + H]^+^, HR-ESI-MS *m/z* 1135.7701 [M + H]^+^ (Calcd for C_57_H_103_N_10_O_13_), LC-MS/MS data see supplementary information (S11). ^1^H-NMR and ^13^C-NMR spectroscopic data see supplementary information (S1).

**Leucinostatin P (3):** 13.6 mg of yellow powder, ${\left[\alpha \right]}_{D}^{20}$ +30 (c 0.5, MeOH); UV (MeOH) λ_max_ (log ε) 222 nm, 193 nm, 223 (sh 4.27). LCMS *m/z* 1121.1 [M + H]^+^, HR-ESI-MS *m/z* 1121.7544 [M + H]^+^ (calcd for C_56_H_101_N_10_O_13_), LC-MS/MS data see supplementary (Figure S18). ^1^H-NMR and ^13^C-NMR are listed in Table S17.

**Arthrichitin D (4):** 0.7 mg of white powder,$\;{\left[\alpha \right]}_{D}^{20}$ +28 (c 0.5, MeOH); UV (MeOH) λ_max_ (log ε) 238 nm, 192 nm, HR-ESI-MS *m/z* 839.4913 (calcd for C_44_H_66_N_6_O_10_). ^1^H-NMR and ^13^C-NMR spectroscopic data see supplementary information (S24).

**Chaetoglobosin B (5):**^1^H-NMR spectroscopic data see supplementary information (S31).

**19-O-acetyl-chaetoglobosin B (6):**^1^H-NMR and ^13^C-NMR spectroscopic data see supplementary information (S32).

### 3.4. Marfay’s Analysis

Leucinostatin P **3** was subjected to acid hydrolysis following Marfay’s protocol in order to determine the absolute configuration of the amino acids leucine and proline [25]. In short, 500 μg of compound 3 was reacted with 1 mL 6 M HCL at 80 °C for 12 h in a heating block. Afterwards, the reaction mixture was dried and dissolved in 120 µL of water to constitute the peptide hydrolysate, which was split in two vials, each with 50 µL, and added to 20 µL NaHCO_3_. 50 µL of Marfay’s reagent, 1-fluoro-2-4-dinitrophenyl-5-l-alanine amide (l-FDAA), prepared as (1%) 10 mg/mL in acetone, were added to each of the two vials. The reaction mixtures were stirred at 37 °C for 60 min and cooled to room temperature. Twenty microliters of 1 M HCl were mixed with the vials contents to stop the reaction and evaporated to dryness. After dilution with methanol (800 µL), the reaction products were analyzed by LC-MS. Standards of d-leucine, dl-leucine, l-proline and d-proline (1 mg each) were dissolved in 500 µL water and reacted with 50 µL of the l-FDAA in parallel with the peptide hydrolysate, to give the derivatized l-FDAA standards. Subsequently, Marfay’s derivatives of compound **3** and the authentic amino acids were detected by HPLC-DAD-MS ion trap mass spectrometer in positive and negative modes.

### 3.5. Mosher Ester Hydrolysis and Absolute Configuration

The S-MTPA ester was prepared by dissolving 0.5 mg of compound **3** in 600 µL of pyridine-*d*_5_, and 10 µL of the R-(−)-MTPA-Cl was added to the vial, which was magnetically stirred for 1 h at room temperature [25,26]. This was followed by the transfer of the whole reaction volume to the NMR tube. Both the ^1^H-NMR and the HSQC spectra were recorded and chemical shift values with considerable changes were noted as shown in Table S2 (Supplementary Information). Exactly the same experimental procedure was repeated to prepare the R-MTPA-methyl ester but with using the S-(+) MTPA-Cl in pyridine-*d*_5_ [27,28].

### 3.6. Nematicidal Activity

All compounds were tested against *P. penetrans and C. elegans* for their nematicidal effects. *Caenorhabditis elegans* was cultivated as described by Ashrafi et al. [10] and Helaly et al. [11]. *Pratylenchus penetrans* was propagated on surface sterilized carrot discs following Elhady et al. [29]. The freshly obtained adults and juveniles of both nematode species were collected and used for the experiments. The number of nematodes (ca. 600 per well) was adjusted to 100/mL in sterile deionized water. The tested compounds were transferred into the wells with a final concentration of 10, 25, 50, and 100 µg/mL using methanol 100 µg/mL as the negative control and ivermectin as the positive control. All samples were measured in triplicates.

### 3.7. Evaluation of Chaetoglobosin B for its Effect on Actin in Mammalian Cells

**Figure 3:**
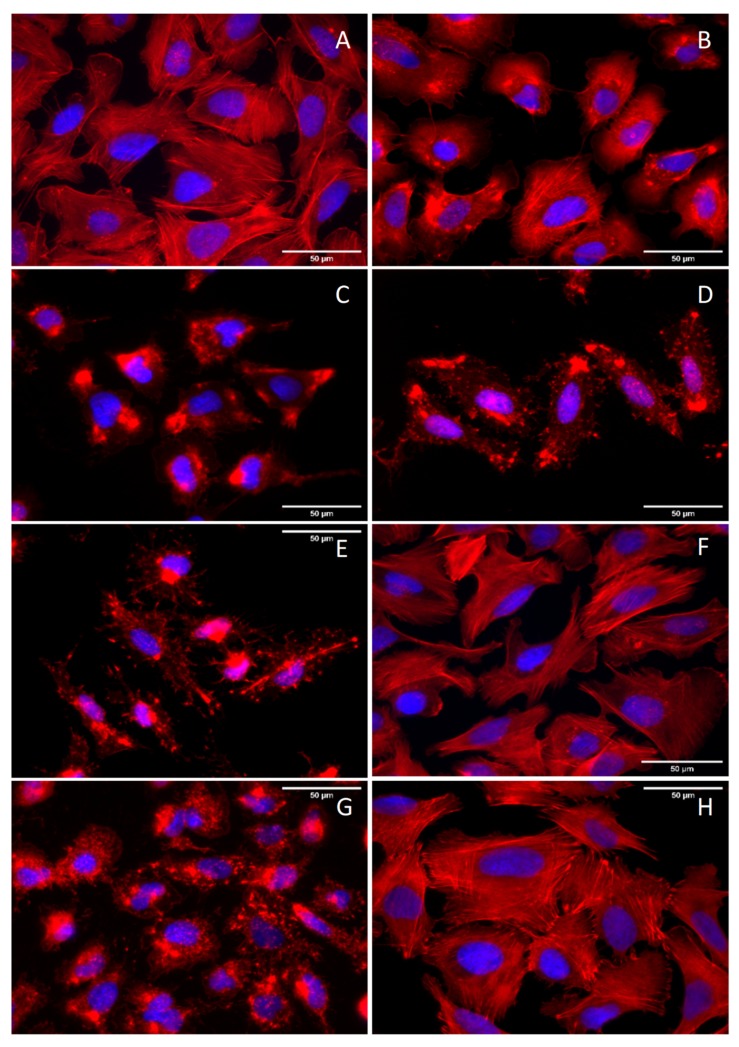
Overlay images of U2OS cells treated with varying concentrations of chaetoglobosin B (**A**–**E,G**) and DMSO as vehicle control (**F,H**) after one hour, the fixed and stained with a fluorescent phalloidin conjugate (Phalloidin–ATTO 594) and DAPI. Concentrations are as followed: 0.01 µg/mL (**A**), 0.1 µg/mL (**B**), 0.25 µg/mL (**C**), 0.5 µg/mL (**D**), 1 µg/mL (**E**), and. 0.5 µg/mL followed by washout with medium for 1 h (**G**) DSMO vehicle control followed by washout with medium for 1 h. (**H**). The phalloidin- signal for F-actin and DAPI staining for the nucleus are displayed in pseudocolours (red and blue, respectively).

The effect of chaetoglobosin B on the actin cytoskeleton of mammalian cells was studied using the same setup as recently described for its congeners, chaetoglobosins A and D, which were isolated from the same fungus, following the methodology described by Kretz et al. [21]. Briefly, the osteosarcoma human cell line, U2OS [ATCC HTB-96] was grown over the Dulbecco’s modified minimum essential medium (DMEM, life Technologies, CA, USA) supplemented with 10% fetal bovine serum, 1% sodium-pyruvate, 1% l-glutamine and 1% minimum essential medium nonessential amino acids (MEM NEAA) at 37 °C and 5% CO_2_. To analyze the effect of chaetoglobosin B on the cytoskeleton, cells were seeded onto fibrinectin coated coverslips and after spreading overnight, cells were treated for 1 h with chaetoglobosin B, diluted in medium, for 1 h at different concentrations as indicated (Figure 3). After cell fixation in 4% paraformaldehyde (AppliChem, Darmstadt, Germany), they were washed with PBS, permeabilized with PBS containing 0.1% Triton X-100 (Hercules, CA, USA) at room temperature for 1 min before re-immersing again in PBS. Fluorescently labelled Phalloidin ATTO 594 (1:200 ATTO-Tec, Siegen, Germany) was used to stain the actin cytoskeleton in PBS for 1 h, and the nucleus was stained with DAPI (Invitrogen, Carlsbad, CA, USA) mixed with Prolong Diamond antifade mountant. Pictures were captured with an inverted microscope (Axio Vert 135 TV, Zeiss, Jena, Germany) equipped with a Coolsnap 4k camera (Photometrics, Tuscon, AZ, USA), operated using Metamorph software (molecularDevices, San Jose, CA, USA),and processed by Image J (NIH, Bethesda, MD, USA).

## Figures and Tables

**Table 1:** Nematicidal activities of **1**–**4** against plant parasitic (*Pratylenchus penetrans*) and free-living (*Caenorhabditis elegans*) nematodes in the microtiter plate assay [8].

Compound	LD_90_ [µg/mL] *C. elegans*	(LD_90_) µg/mL *P. penetrans*
Leucinostatin U (**1**)	5	100
Leucinostatin Q (**2**)	7	100
Leucinostatin P (**3**)	7	100
Arthrichitin D (**4**)	100	>100
19-*O*-acetyl-chaetoglobosin B (**6**)	25	>100
Ivermectin (positive control)	1	10

## Supplementary Materials

Supplementary Materials are available online at https://www.mdpi.com/2079-6382/9/3/132/s1. Figure S1: ^1^H-NMR and ^13^C-NMR data of leucinostatins 1 and 2 and the characteristic chemical shift values. Figure S2: HR-ESI-MS-MS spectra of leucinostatin U 1. Figure S3: HR-ESI-MS spectra of Marfay’s reaction products of leucinostatin U 1. Figure S4: ∆δSR chemical shifts of MTPA (α-methoxy-α-trifluoromethylphenylacetic acid) (Mosher) derivatives of the proline, AHMOD and Hy-Leu of leucinostatin U 1. Table S5: ^1^H-NMR data of leucinostatin U 1 and the characteristic chemical shift value differences ∆δSR of the corresponding Mosher esters, recorded at 700 MHz (75% CH_3_CN/D_2_O). Figure S6: HSQC spectra of leucinostatin U 1. Figure S7: HMBC spectra of leucinostatin U 1. Figure S8: COSY spectra of leucinostatin U 1. Figure S9: TOCSY spectra of leucinostatin U 1. Figure S10: NOESY spectra of leucinostatin U 1. Figure S11: HR-ESI-MSMS of leucinostatin Q 2. Figure S12: HSQC spectra of leucinostatin Q 2. Figure S13: HMBC spectra of leucinostatin Q 2. Figure S14: COSY spectra of leucinostatin Q 2. Figure S15: TOCSY spectra of leucinostatin Q 2. Figure S16: NOESY spectra of leucinostatin Q 2. S17 Table: ^1^H-NMR and ^13^C-NMR data of leucinostatin P 3 and its characteristic chemical shift values. Figure S18: HR-ESI-MSMS fragmentation pattern of leucinostatin P 3. Figure S19: HSQC spectra of leucinostatin P 3. Figure S20: HMBC spectra of leucinostatin P 3. Figure S21: COSY spectra of leucinostatin P 3. Figure S22: TOCSY spectra of leucinostatin P 3. Figure S23: NOESY spectra of leucinostatin P 3. Table S24: ^1^H-NMR and ^13^C-NMR data of arthrichitin D 4 and its characteristic chemical shift values. Figure S25: HR-ESI-MS of arthrichitin D 4. Figure S26: HSQC spectra of arthrichitin D 4. Figure S27: HMBC spectra of arthrichitin D 4. Figure S28: COSY spectra of arthrichitin D 4. Figure S29: TOCSY spectra of arthrichitin D 4. Figure S30: NOESY spectra of arthrichitin D 4. Text S31: ^1^H-NMR and ^13^C-NMR data of chaetoglobosin B 5 and its characteristic chemical shift values. Text S32: ^1^H-NMR and ^13^C-NMR data of 19-*O*-acetyl chaetoglobosin B 6 and its characteristic chemical shift values.

## References

[1] Li G., Zhang K., Xu J., Dong J., Liu Y. (2007). Nematicidal substances from fungi. Recent Pat. Biotechnol..

[2] Jones J.T., Haegeman A., Danchin E.G., Gaur H.S., Helder J., Jones M.G., Kikuchi T., Manzanilla-Lopez R., Palomares-Rius J.E., Wesemael W.M. (2017). Top 10 plant-parasitic nematodes in molecular plant pathology. Mol. Plant Pathol..

[3] Jones M.G.K., Nyarko J.F. (2014). Molecular biology of root lesion nematodes (*Pratylenchus* spp.) and their interaction with host plants. Ann. Appl. Biol..

[4] Hyde K.D., Xu J., Rapior S., Jeewon R., Lumyong S., Niego A.G.T., Abeywickrama P.D., Aluthmuhandiram J.V.S., Brahamanage R.S., Brooks S. (2019). The amazing potential of fungi: 50 ways we can exploit fungi industrially. Fungal Divers..

[5] Yang J., Stadler M., Chuang W., Wu S., Ariyawansa H.A. (2020). In vitro inferred interactions of selected entomopathogenic fungi from Taiwan and eggs of *Meloidogyne graminicola*. Mycol. Prog..

[6] Degenkolb T., Vilcinskas A. (2016). Metabolites from nematophagous fungi and nematicidal natural products from fungi as an alternative for biological control. Part I: Metabolites from nematophagous ascomycetes. Appl. Microbiol. Biotechnol..

[7] Mayer A., Anke H., Sterner O. (1997). Omphalotin, a new cyclic peptide with potent nematicidal activity from *Omphalotus olearius* I. Fermentation and biological activity. Nat. Prod. Lett..

[8] Ashrafi S., Stadler M., Dababat A.A., Richert-Pöggeler K.R., Finckh M.R., Maier W. (2017). *Monocillium gamsii* sp. nov. and *Monocillium bulbillosum*: Two nematode-associated fungi parasitising the eggs of *Heterodera filipjevi*. MycoKeys.

[9] Ashrafi S., Knapp D.G., Blaudez D., Chalot M., Maciá-Vicente J.G., Zagyva I., Dababat A.A., Maier W., Kovács G.M. (2018). Inhabiting plant roots, nematodes, and truffles—*Polyphilus*, a new helotialean genus with two globally distributed species. Mycologia.

[10] Ashrafi S., Helaly S., Schroers H.J., Stadler M., Richart-Poeggeler K.R., Dababat K.A., Maier W. (2017). *Ijuhya vitellina* sp. *nov*, a novel source for chaetoglobosin A, is a destructive parasite of the cereal cyst nematode *Heterodera filipjevi*. PLoS ONE.

[11] Helaly S.E., Ashrafi S., Teponno R.B., Bernecker S., Dababat A.A., Maier W., Stadler M. (2018). Nematicidal cyclic lipodepsipeptides and a xanthocillin derivative from a phaeosphariaceous fungus parasitizing eggs of the plant parasitic nematode *Heterodera filipjevi*. J. Nat. Prod..

[12] Momose I., Onodera T., Doi H., Adachi H., Iijima M., Yamazaki Y., Sawa R., Kubota Y., Igarashi M., Kawada M. (2019). Leucinostatin Y: A peptaibiotic produced by the entomoparasitic fungus *Purpureocillium lilacinum* 40-H-28. J. Nat. Prod..

[13] Vertuani G., Boggian M., Scatturin A., Ricci M., Meli Balbocchino B., Tuttobello L., Rossi C. (1995). Structure activity studies on chemically modified homologues of the antibiotic phytotoxic leucinostatin A. J. Antibiot..

[14] Vijayakumar E.K.S., Roy K., Chatterjee S., Deshmukh S.K., Ganguli B.N., Fehlhaber H.W., Kogler H. (1996). Arthrichitin. A new cell wall active metabolite from *Arthrinium phaeospermum*. J. Org. Chem..

[15] Arai T., Mikami Y., Fukushima K., Utsumi T., Yazawa K. (1973). A new antibiotic, leucinostatin, derived from *Penicillium lilacinum*. J. Antibiot..

[16] Harrington J.M. (2012). Antimicrobial peptide killing of African trypanosomes. Parasite Immunol..

[17] Csermely P., Radics L., Rossi C., Szamel M., Ricci M., Mihály K., Somogyi J. (1994). The nonapeptide leucinostatin A acts as a weak ionophore and as an immunosuppressant on T lymphocytes. Biochim. Biophys. Acta.

[18] Park J.O., Hargreaves J.R., Mcconville E.J., Stirling G.R., Ghisalberti E.L., Sivasithamparam K. (2004). Production of leucinostatins and nematicidal activity of Australian isolates of *Paecilomyces lilacinus* (Thom) Samson. Lett. Appl. Microbiol..

[19] Degenkolb T., Gams W., Brückner H. (2008). Natural cyclopeptaibiotics and related cyclic tetrapeptides: Structural diversity and future prospects. Chem. Biodivers..

[20] Shima A., Fukushima K., Arai T., Terada H. (1990). Dual inhibitory effects of the peptide antibiotics leucinostatins on oxidative phosphorylation in mitochondria since leucinostatin. Cell Struct. Funct..

[21] Kretz R., Wendt L., Wongkanoun S., Luangsa-Ard J.J., Surup F., Helaly S.E., Noumeur S.R., Stadler M., Stradal T.E.B. (2019). The effect of cytochalasans on the actin cytoskeleton of eukaryotic cells and preliminary structure–activity relationships. Biomolecules.

[22] Yahara I., Harada F., Sekita S., Yoshihira K., Natori S. (1982). Correlation between effects of 24 different cytochalasins on cellular structures and cellular events and those on actin in vitro. J. Cell Biol..

[23] Scherlach K., Boettger D., Remme N., Hertweck C. (2010). The chemistry and biology of cytochalasans. Nat. Prod. Rep..

[24] Li H., Xiao J., Gao Y.Q., Tang J.J., Zhang A.L., Gao J.M. (2014). Chaetoglobosins from *Chaetomium globosum*, an endophytic fungus in *Ginkgo biloba*, and their phytotoxic and cytotoxic activities. J. Agric. Food Chem..

[25] Surup F., Chauhan D., Niggemann J., Bartok E., Herrmann J., Keck M., Zander W., Stadler M., Hornung V., Müller R. (2018). Activation of the NLRP3 Inflammasome by hyaboron, a new asymmetric boron-containing macrodiolide from the Myxobacterium *Hyalangium minutum*. ACS Chem. Biol..

[26] Schlingmann G., Milne L., Williams D.R., Carter G.T. (1998). Cell wall active antifungal compounds produced by the marine fungus *Hypoxylon oceanicum* LL-15G256 II. J. Antibiot..

[27] Hoye T.R., Jeffrey C.S., Shao F. (2007). Mosher ester analysis for the determination of absolute configuration of stereogenic (chiral) carbinol carbons. Nat. Protoc..

[28] Surup F., Pommerehne K., Schroers H.J., Stadler M. (2018). Elsinopirins A–D, decalin polyketides from the ascomycete *Elsinoë pyri*. Biomolecules.

[29] Elhady A., Giné A., Topalovic O., Jacquiod S., Sørensen S.J., Sorribas F.J., Heuer H. (2017). Microbiomes associated with infective stages of root-knot and lesion nematodes in soil. PLoS ONE.

