# Supplementary material for: New Peptaibiotics and a Cyclodepsipeptide from Ijuhya vitellina: Isolation, Identification, Cytotoxic and Nematicidal Activities
Source: Antibiotics (Basel). 2020 Mar 22;9(3):132. doi: 10.3390/antibiotics9030132 (PMC7148537; doi:10.3390/antibiotics9030132)
Supplement: Supplementary file 1 [file antibiotics-09-00132-s001.pdf]

Supporting Information

New peptaibiotics and a cyclodepsipeptide from *Ijuhya vitellina:* Isolation, identification, cytotoxic and nematicidal activities.

Ashaimaa Y. Moussa[1,2], Christopher Lambert[3], Theresia E.B. Stradal[3], Samad Ashrafi[4], Wolfgang Maier[4] , Marc Stadler[1], Soleiman Helaly[1,5]*

[1] Department of Microbial Drugs, Helmholtz Centre for Infection Research, Inhoffenstraße 7, 38124 Braunschweig, Germany.

[2] Department of Pharmacognosy, Faculty of Pharmacy, Ain shams University, Abbassia, 11566, Cairo, Egypt.

[3] Department of Cell Biology, Helmholtz Centre for Infection Research (HZI), Inhoffenstraße 7, 38124, Braunschweig, Germany.

[4] Institute for Epidemiology and Pathogen Diagnostics, Julius Kühn-Institut (JKI)– Federal Research Centre for Cultivated Plants, Braunschweig, Germany.

[5] Department of Chemistry, Faculty of Science, Aswan University, Aswan, Egypt. *Correspondence: soleiman.helaly@helmholtz-hzi.de.

Contents

LIST OF TABLES iv
LIST OF FIGURES v
LIST OF TEXTS..vi
1 and 2D NMR spectral data for Leucinostatin U 1 3
1 and 2D NMR spectral data for leucinostain Q 2..14
1 and 2D NMR spectral data for leucinostain P 3..24
1 and 2D NMR spectral data for arthrichitin D 4 34
1D NMR data for chaetoglobosin B 5 41
1D NMR data for 19-O-acetyl.chaetoglobosin B 6..41

LIST OF TABLES

S1 Table: ^{1}H NMR and ^{13}C NMR data of leucinostatins 1 & 2 and the characteristic chemical shift values……………………………………………………………………………………………………...…...1
S2 Table: ^{1}H NMR data of leucinostatin U 1 and the characteristic chemical shift value differences ΔδSR of the corresponding Mosher esters, recorded at 700 MHz (75% CH_3CN/D_2O)……………….....13
S3 Table: ^{1}H NMR and ^{13}C NMR data of leucinostatin P 3 and its characteristic chemical shift values……………………………………………………………………………………………………......22
S4 Table: ^{1}H NMR and ^{13}C NMR data of arthrichitin D 4 and its characteristic chemical shift values.32

LIST OF FIGURES

S1 figure: ^{1}HNMR spectra of leucinostatin U 1……………………………………………………..…3
S2. Figure: ^{13}CNMR spectra of leucinostatin U 1…………………………………………………....4
S3 Figure: HSQC spectra of leucinostatin U 1……………………………………………………..…5
S4 Figure: HMBC spectra of leucinostatin U 1………………………………………………………6
S5 Figure: COSY spectra of leucinostatin U 1…………………………………………………..……7
S6 Figure: NOESY spectra of leucinostatin U 1………………………………………………………8
S7 Figure: TOCSY spectra of leucinostatin U 1……………………………………………………..…9
S8 Figure: HR-ESI-MS spectra of Marfay's reaction products of leucinostatin U 1…………………10
S9 Figure: ΔδSR chemical shifts of MTPA (α-methoxy-α-trifluoromethylphenylacetic acid) (Mosher) derivatives of the proline, AHMOD and Hy-Leu of leucinostatin U 1…………………………..……11
S10 Figure: HR-ESI-MS-MS fragmentation pattern of leucinostatin U 1………………………….……12
S11 figure: ^{1}HNMR spectra of leucinostatin Q 2…………………………………………………….……14
S12. Figure: ^{13}CNMR spectra of leucinostatin Q 2……………………………………………….……15
S13 Figure: HSQC spectra of leucinostatin Q 2……………………………………………………..…16
S14Figure: HMBC spectra of leucinostatin Q 2……………………………………………………….…..17
S15 Figure: COSY spectra of leucinostatin Q 2…………………………………………………………18
S16 Figure: NOESY spectra of leucinostatin Q 2……………………………………………………..…..19
S17 Figure: TOCSY spectra of leucinostatin Q 2……………………………………………………......20
S18 Figure: HR-ESI-MS-MS fragmentation pattern of leucinostatin Q 2……………………………..21
S19 figure: ^{1}HNMR spectra of leucinostatin P 3………………………………………………………..…24
S20 Figure: HSQC spectra of leucinostatin P 3………………………………………………………....25
S21 Figure: HMBC spectra of leucinostatin P 3…………………………………………………………26
S22 Figure: COSY spectra of leucinostatin P 3………………………………………………………..…27
S23 Figure: NOESY spectra of leucinostatin P 3………………………………………………………...28
S24 Figure: TOCSY spectra of leucinostatin P 3……………………………………………………..…29
S25 Figure: HR-ESI-MSMS fragmentation pattern of leucinostatin P 3……………………………..30
S26 Figure: HR-ESI-MS-MS fragments of leucinostatin P 3……………………………………….....31
S27.Figure: ^{1}HNMR spectra of arthrichitin D 4…………………………………………………………....34
S28 Figure: ^{13}CNMR spectra of arthrichitin D 4…………………………………………..……....35
S29 Figure: HSQC spectra of arthrichitin D 4…………………………………………………………..36
S30 Figure: HMBC spectra of arthrichitin D 4…………………………………………………………..37
S31 Figure: COSY spectra of arthrichitin D 4…………………………………………………………...38
S32 Figure: NOESY spectra of arthrichitin D 4…………………………………………………………39
S33 Figure: ROESY spectra of arthrichitin D 4…………………………………………………………..40
S34 Figure: HR-ESI-MS of arthrichitin D 4. ……………………………………………………………..41

LIST OF TEXT:

S1 Text: ^{1}H NMR spectral data of chaetoglobosin B (5) and its characteristic chemical shift values….42
S2 Text: ^{1}H NMR and ^{13}C NMR data of 19-O-acetyl chaetoglobosin B (6) and its characteristic chemical shift values………………………………………………………………………………………………..42

S1 Table. ^{1}H NMR and ^{13}C NMR data of **1 & 2** and the characteristic chemical shift values.

		**leucinostatin U (1)**		**leucinostatin Q (2)**	
**residue**	position	δC, type	δH(J in Hz)	δC, type	δH(J in Hz)
**ΔMHA**	1	165.15, C		165.45,C	
	2	120.27, CH	6.14, (15.04)	120.65,CH	6.25
	3	150.02,CH	6.58 (7.7, 15.0)	151.24,CH	6.66
	4	38.10,CH	2.22	37.82,CH	2.26
	5	29.23,CH2	1.36	28.86,CH2	1.38
	6	12.10,CH3	0.88	11.88,CH3	0.87
	7	19.86,CH3	0.99(6.3)	18.85,CH3	1.04
**Pro**	1	174.76,C		173.6,C	
	2	62.28,CH	4.2(4.12, 8.9)	61.61,CH	4.26
	3	30.22,CH2	2.22	29.84,CH2	2.19
			1.8		1.82
	4	24.80,CH2	1.94	24.80,CH2	1.94
	5	47.75,CH2	3.91	47.53,CH2	3.81
			3.52		3.6
**AHMOD**	1	175.05, C		173.5,C	
	2	54.38,CH	4.09	52.84,CH	4.17
	3	34.82, CH2	2.07	36.01,CH2	1.88
			1.16		1.36
	4	25.47,CH	1.76	26.18,CH	1.68
	5	46.19,CH2	1.25	45.58,CH2	1.29
			1.17		1.24
	6	64.23,CH	3.94	64.86,CH	3.97
	7	50.98,CH2	2.39(3.4,14.3)	50.57,CH2	2.42
			2.33(8.7,14.3)		
	8	210.41,C		210.12,C	
	9	36.14,CH2	2.42	36.26,CH2	2.44
	10	7.89,CH3	0.89(7.4)	7.79,CH3	0.9
	11	22.91,CH3	0.89	23.32,CH3	0.9
	NH		9.68		6.97
**Hyleu**	1	172.10,C		172.16	
	2	61.77,CH	3.81	58.64,CH	4.11
	3	73.24,CH	3.76	75.05,CH	3.64
	4	29.67,CH	1.59	30.34,CH	1.58
	5	15.13,CH3	0.88	17.48,CH3	0.91
	6	19.88,CH3	0.79	19.70,CH3	0.83
	NH		9.03		7.74
**Aib1**	1	174.56,C		174.6	

	2	55.7,C		55.80,C	
	3	22.30,CH3	1.34	23.39,CH3	1.38
	4	26.10,CH3	1.41	25.14,CH3	1.34
	NH		8.47		8.16
**Aib2**	1	175.64,C		173.24,C	
	2	56.0,C		55.94,C	
	3	22.6,CH3	1.32	25.63,CH3	1.31
	4	25.83,CH3	1.46	23.32,CH3	1.38
	NH		7.56		7.43
**Leu**	1	174.43,C		173.16,C	
	2	55.82,CH	3.78	53.76,CH	3.83
	3	39.80,CH2	1.75	39.41,CH2	1.77
			1.64		1.58
	4	24.54,CH	1.76	24.55,CH	1.76
	5	22.57,CH3	0.84(6.3)	21.75,CH3	0.84
	6	22.91,CH3	0.89	23.32,CH3	0.9
	NH		7.75		6.92
**Aib3**	1	173.97,C		175.2,C	
	2	56.04,C		56.09,C	
	3	22.3,CH3	1.36	22.90,CH3	1.38
	4	26.54,CH3	1.4	25.66,CH3	1.35
	NH		8.12		7.57
**Aib4**	1	173.90,C		173.31,C	
	2	55.73,C		55.97,C	
	3	26.79,CH3	1.47	25.76,CH3	1.34
	4	22.90,CH3	1.38	23.44,CH3	1.37
	NH		7.16		7.57
**C-terminal**	1	40.64,CH	4.4(m)	43.15,CH	3.81
	2	68.65,CH2	3.61(9.8)	63.31,CH2	2.31
			3.5(7.6)		
	3	66.59,CH2	3.46 (5.3)	60.21,CH2	2.45
	4	55.48,CH2	3.86 (5.3)	59.22,CH2	3.44
	5	51.91,CH3	3.11(5.9)	19.27,CH3	1.02
	6	51.91,CH3	3.11(5.9)		
	7	20.86,CH3	1.15(6.4)		
	NH		7.36		7.75
	N-CH3			43.41,CH3	2.2

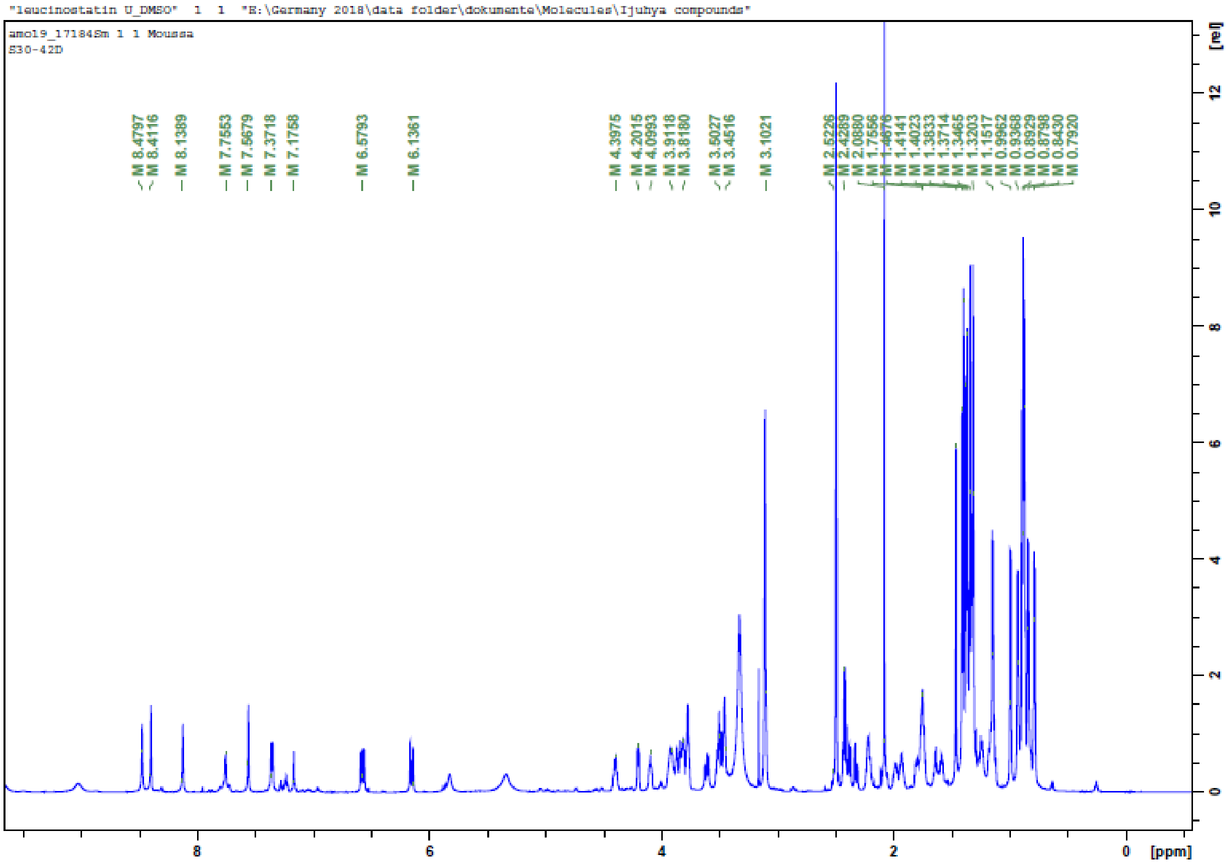

S1 figure: ^{1}HNMR spectra of **leucinostatin U 1.**

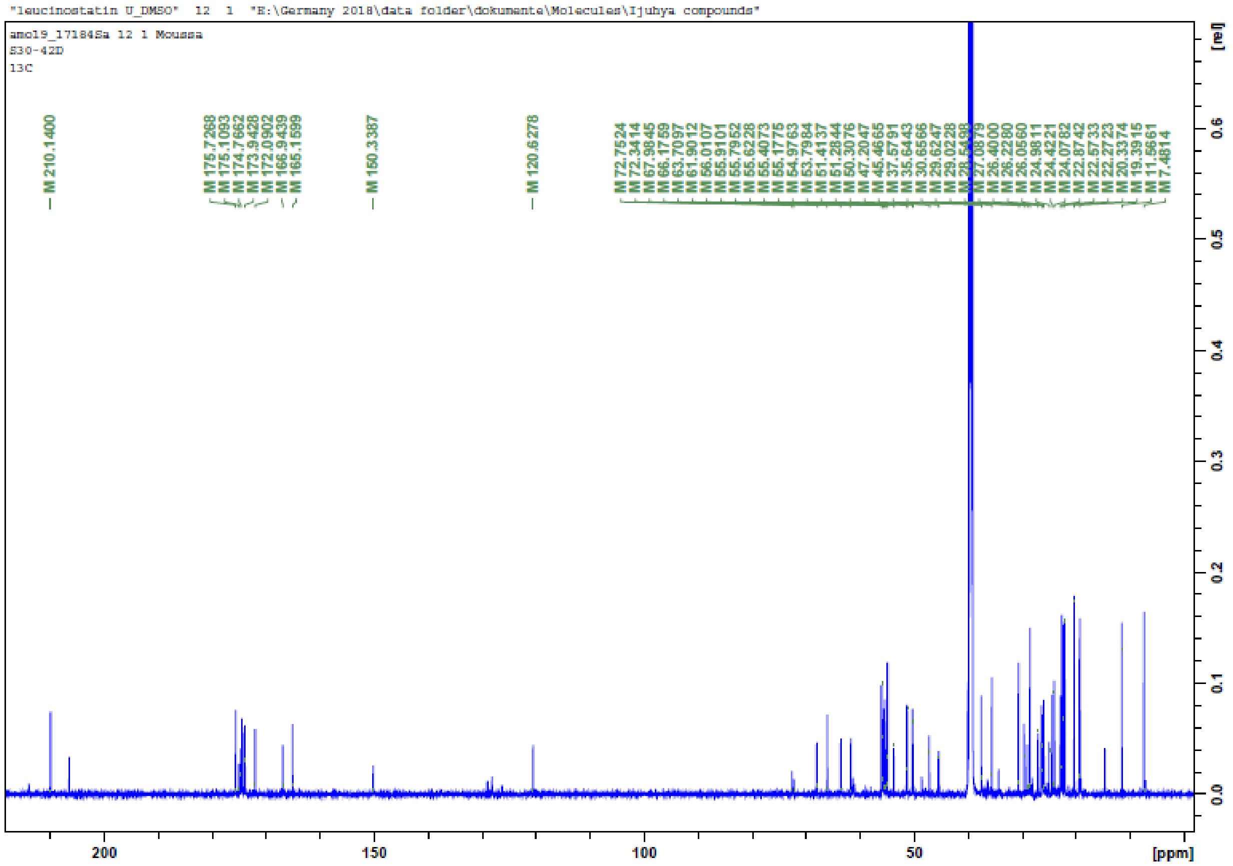

S2. Figure: ^{13}CNMR spectra of **leucinostatin U.1.**

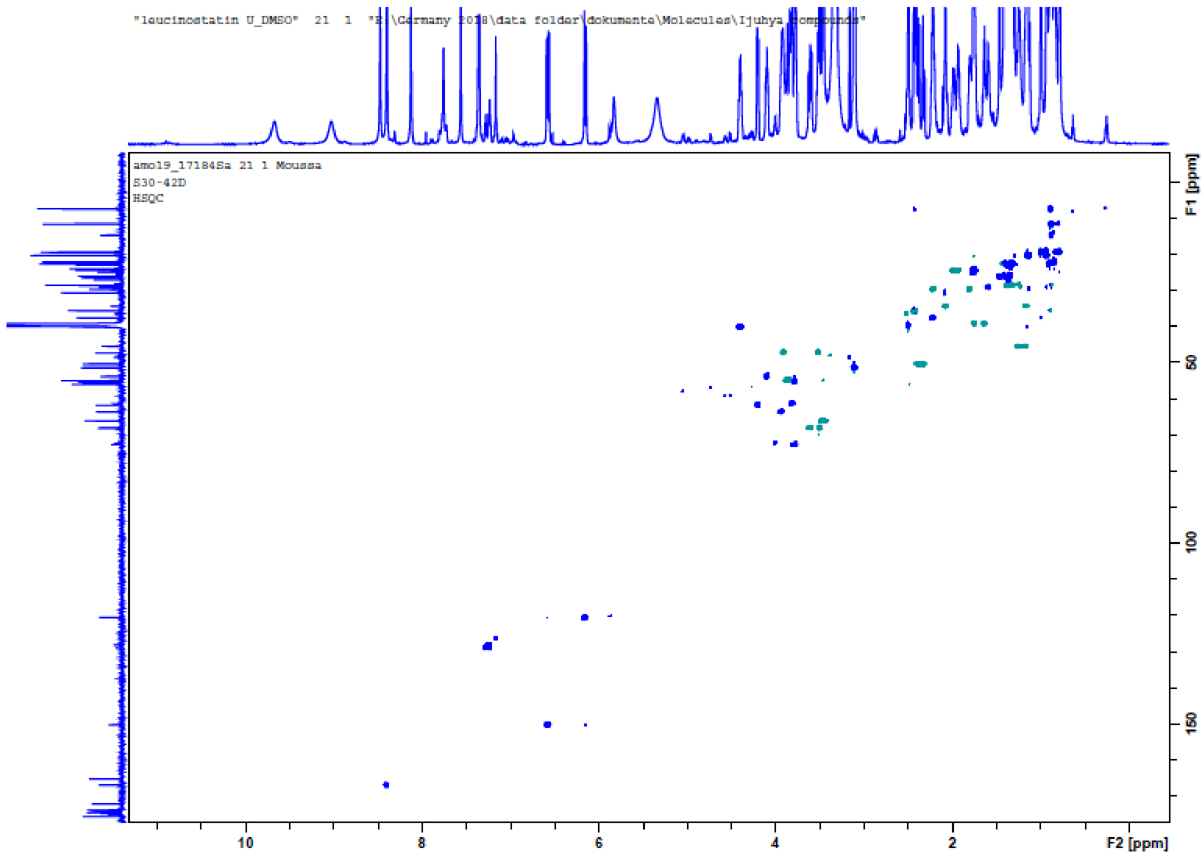

S3 Figure: HSQC spectra **of leucinostatin U 1.**

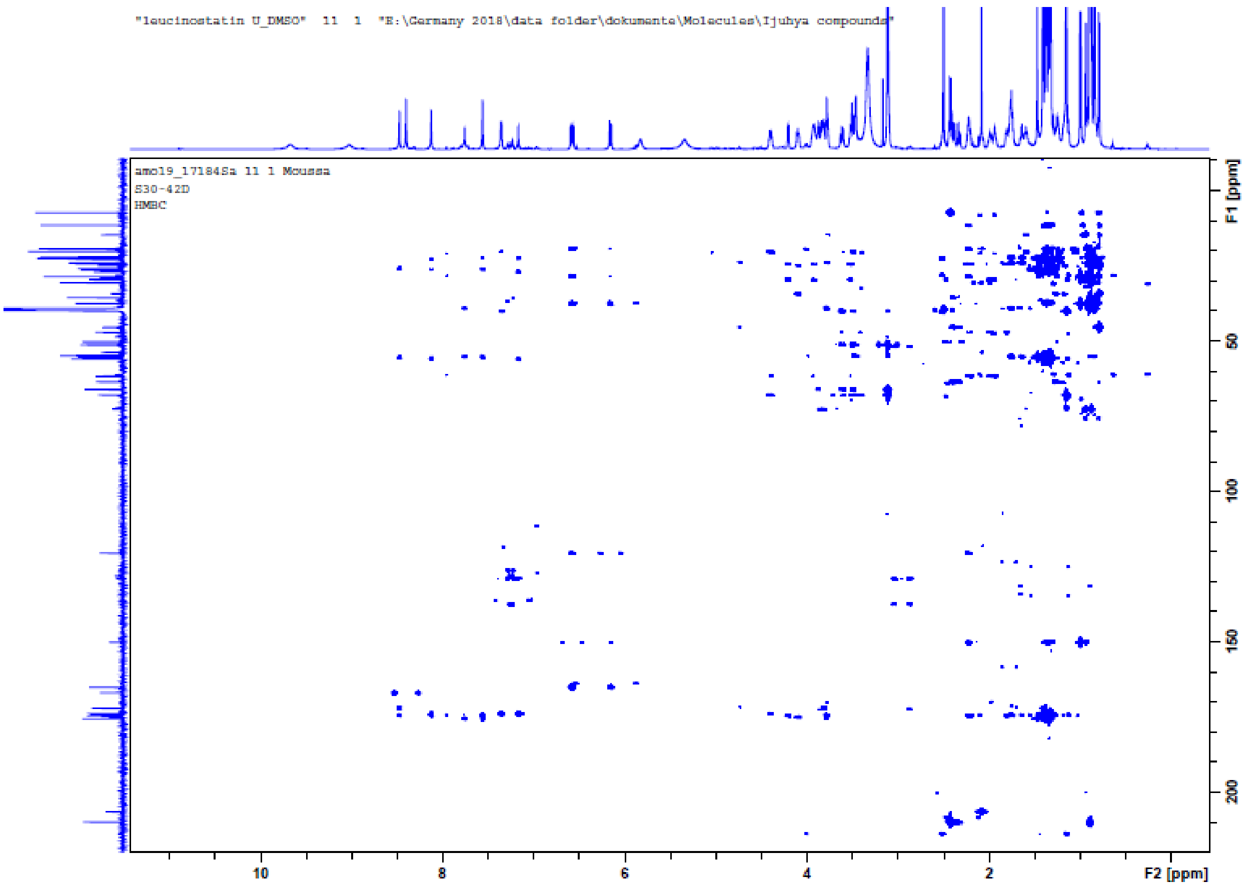

S4 Figure: HMBC spectra **of leucinostatin U 1**

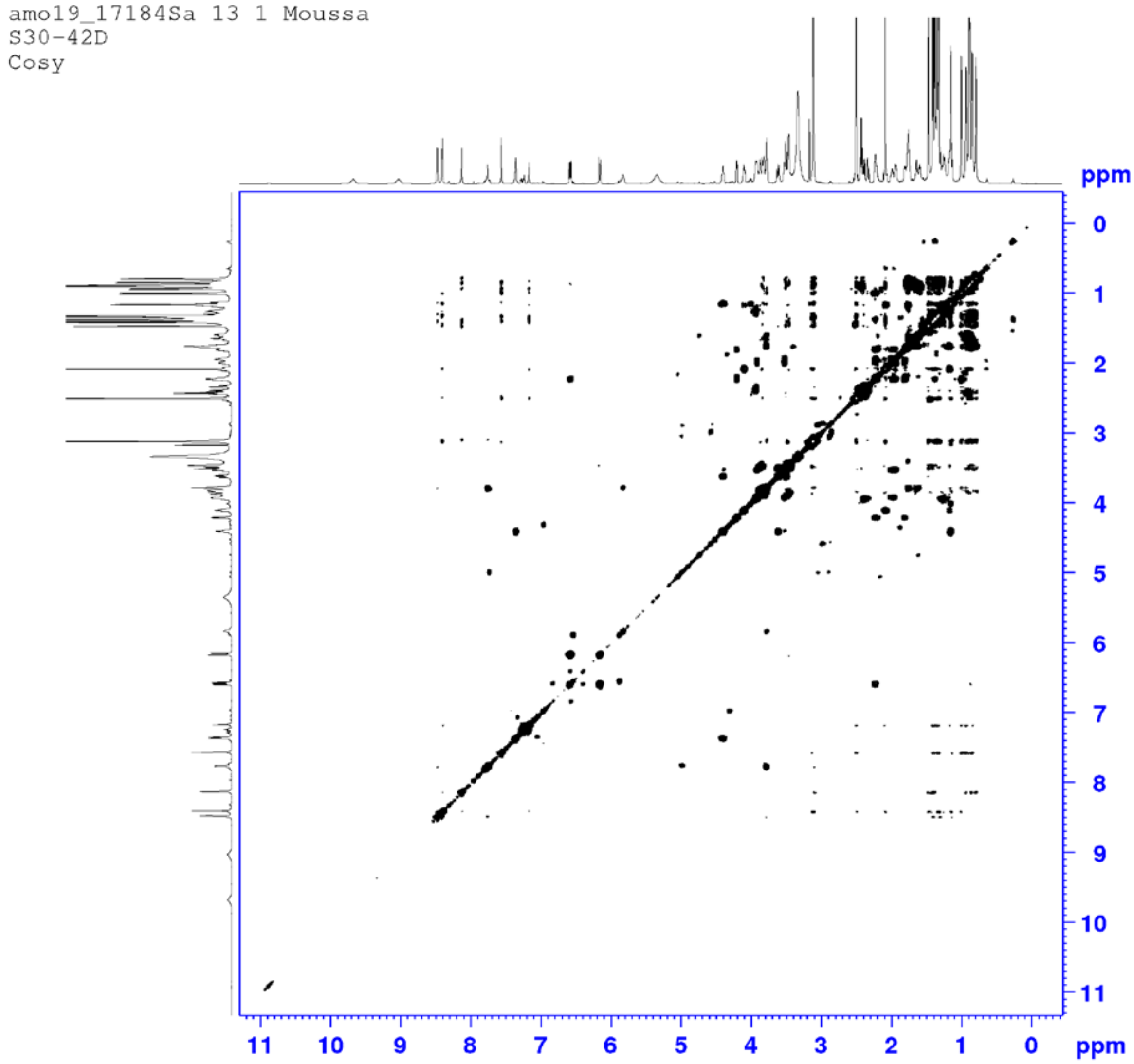

S5 Figure: COSY spectra **of leucinostatin U 1.**

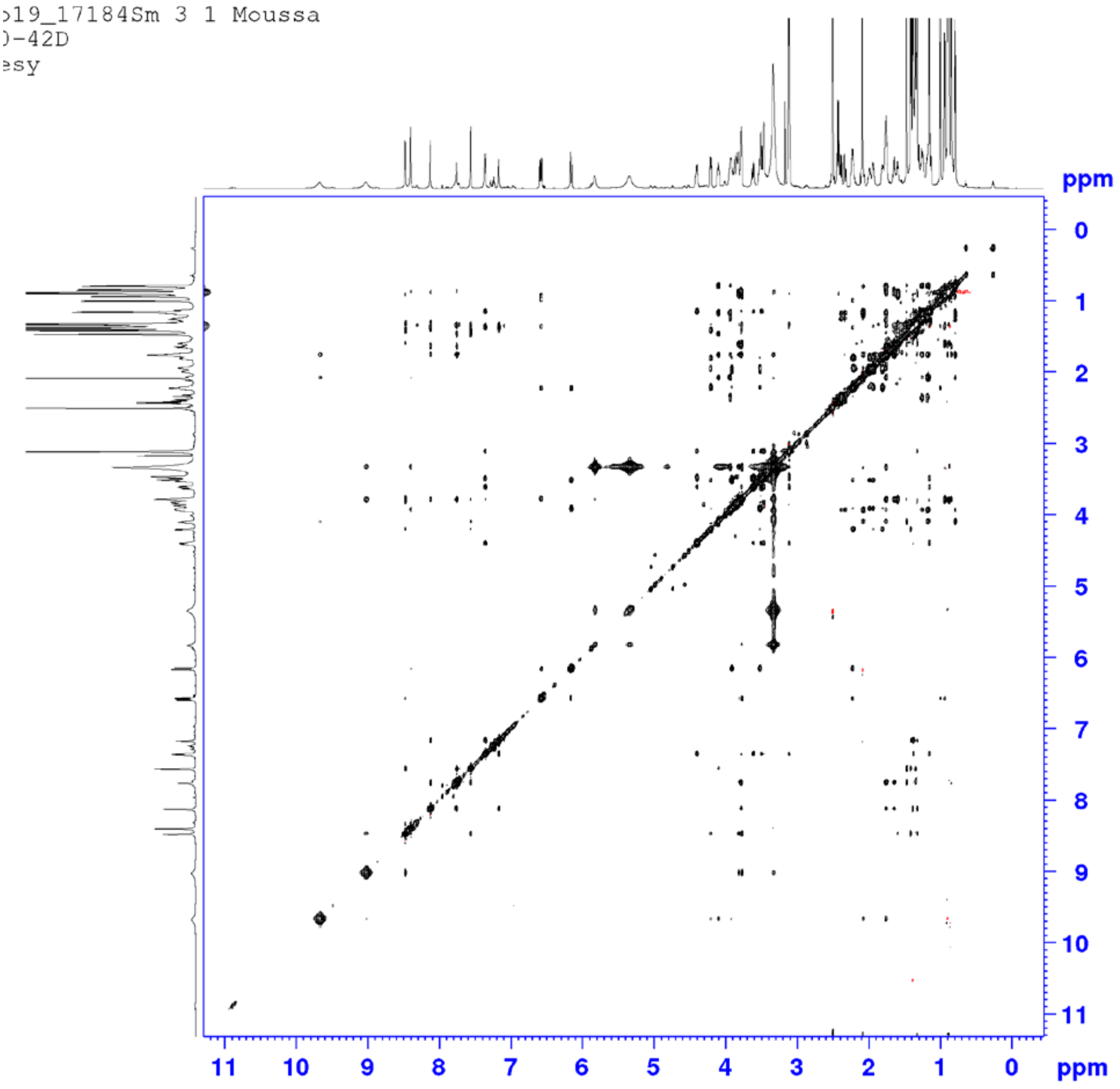

S6 Figure: NOESY spectra **of leucinostatin U 1**

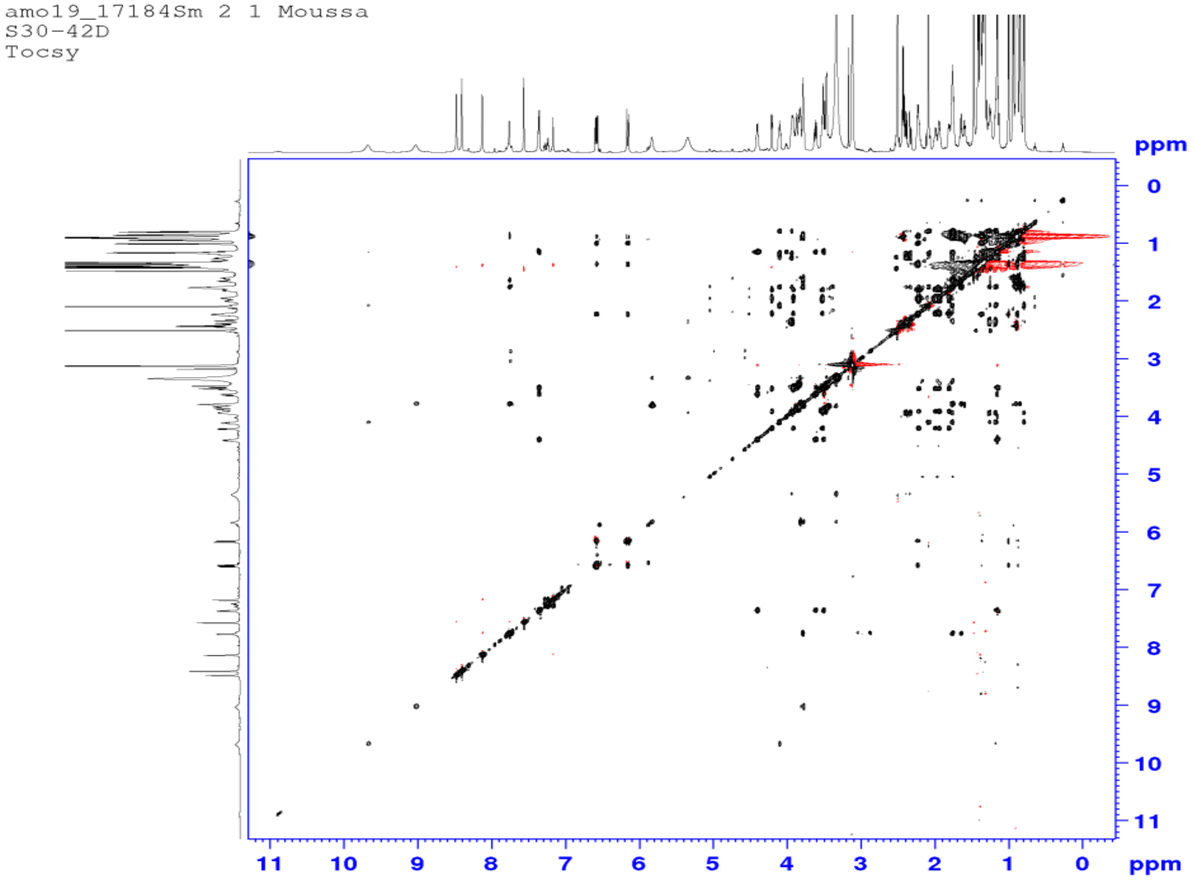

S7 Figure: TOCSY spectra **of leucinostatin U 1.**

.

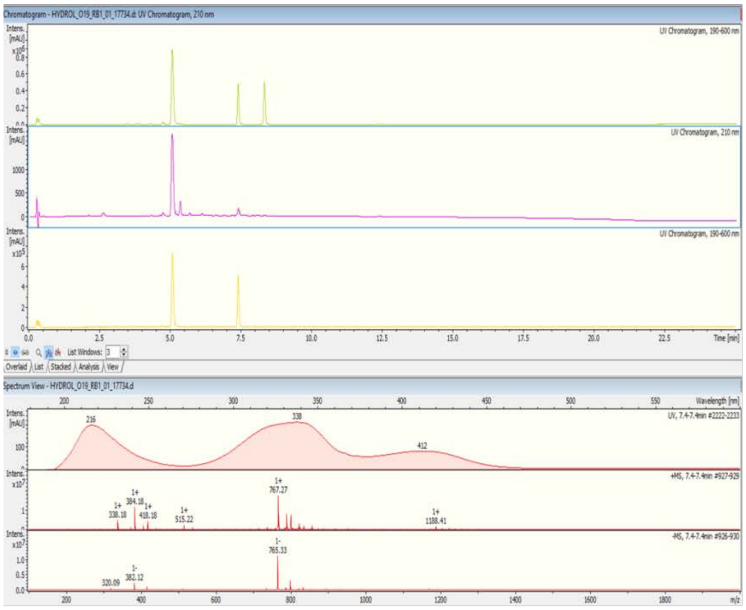

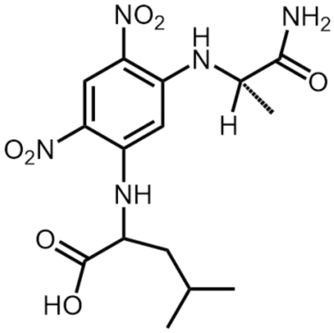

Chemical Formula: C15H21N5O7
Exact Mass: 383,14
Molecular Weight: 383,36

FDLA- D-leucine and DL-leucine

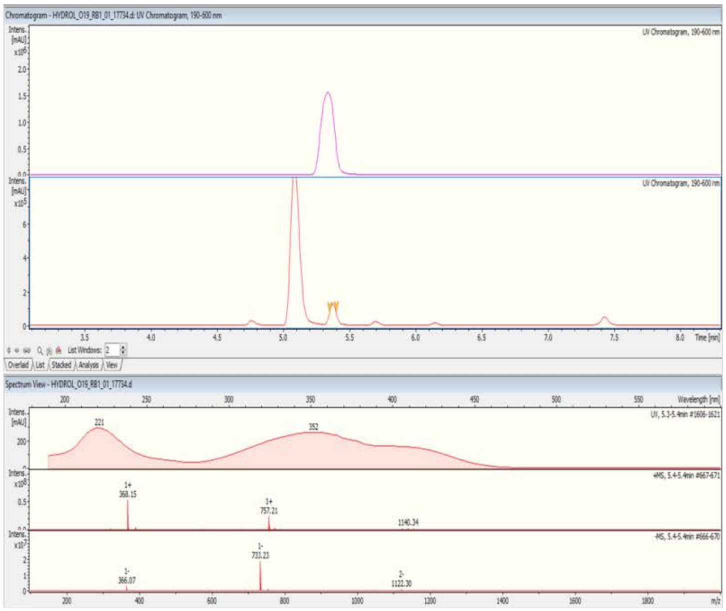

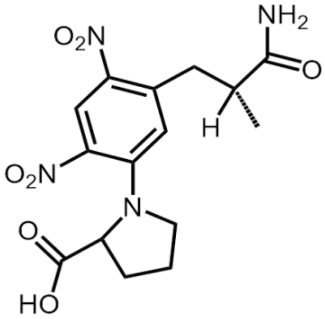

Chemical Formula:
C15H18N4O7
Exact Mass: 366,12
Molecular Weight: 366,33

FDLA-L- Proline and D-proline

S8 Figure. HR-ESI-MS of Marfay's reaction products of **leucinostatin U 1.**

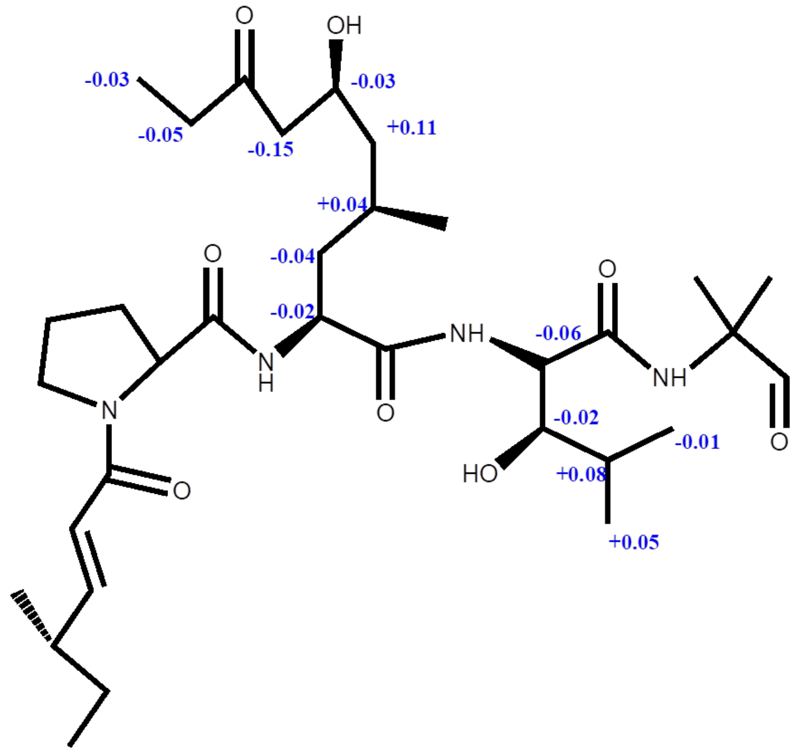

S9 Figure. ΔδSR chemical shifts of MTPA (α-methoxy-α-trifluoromethylphenylacetic acid) (Mosher) derivatives of the proline, AHMOD and Hy-Leu of **1.**

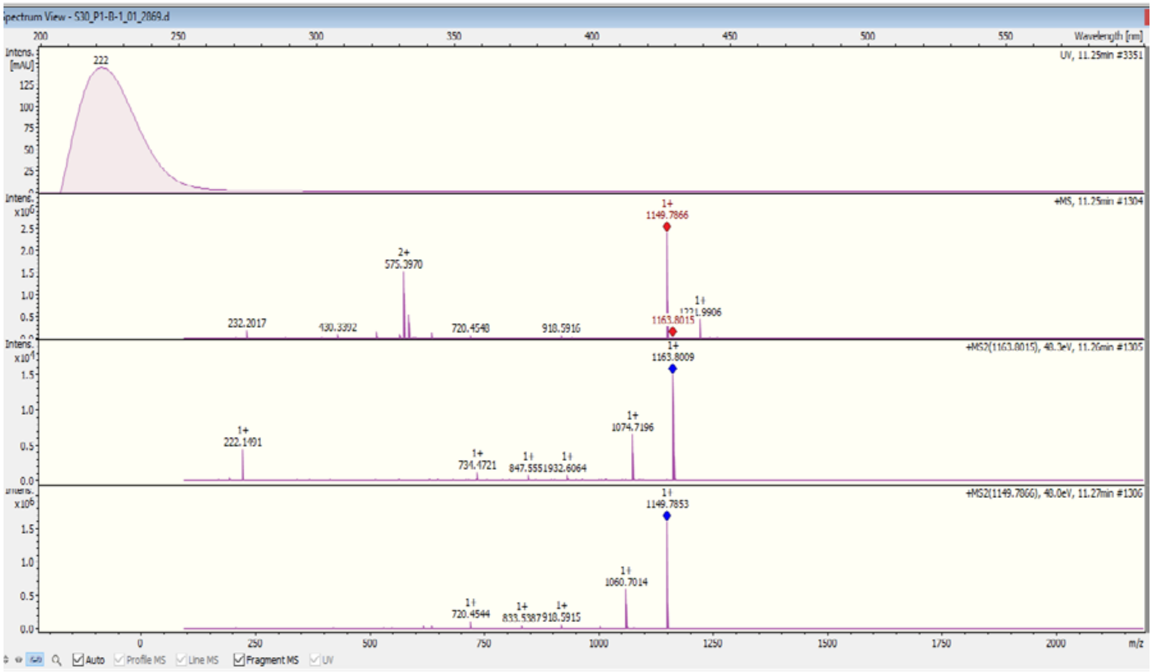

S10 Figure. HR-ESI-MS-MS fragmentation pattern of leucinostatin U **1**.

S2.Table. 1H NMR data of **leucinostatin U 1** and the characteristic chemical shift value differences ΔδSR of the corresponding Mosher esters, recorded at 700 MHz (75% CH3CN/D2O).

position	δH leucinostatin U (1)	δH of leucinostatin U (1) reacted with R-MTPA-Cl	δH of leucinostatin U (1) reacted with S-MTPA-Cl	ΔS-ΔR(ppm)	ΔS-ΔR (Hz)
**AHMOD**					
**2**	4.67	4.73,CH	4.71,CH	-0.02	-14
**3**	2.57, 1.29	1.52, 2.51CH2	1.92, 2.76,CH2	-0.04	-28
**4**	1.74	1.72	1.68, CH2	0.04	28
**10**	1	0.98	0.97	0.01	7
**5**	1.55, 1.19	1.62, 1.30	1.73	0.11	77
**6**	4.58	4.68	4.51,CH2	-0.03	-21
**7**	2.98	2.56, 2.72	3.22, 2.99, CH2	-0.15	-105
**8**	2.57, 1.29	2.64, 2.53	2.59,2.45,CH2	-0.05	-35
**9**	1.06	0.99	1.02	-0.03	-21
**Hyleu**					
**2**	4.39	4.60,CH	4.55,CH	-0.06	-42
**3**	4.52	4.45,CH	4.47,CH	-0.02	-14
**4**	1.88	1.86,CH	1.94,CH	0.08	56
**5**	0.85	0.88,CH3	0.93,CH3	0.05	35
**6**	1.42	1.34,CH3	1.35,CH3	-0.01	-1

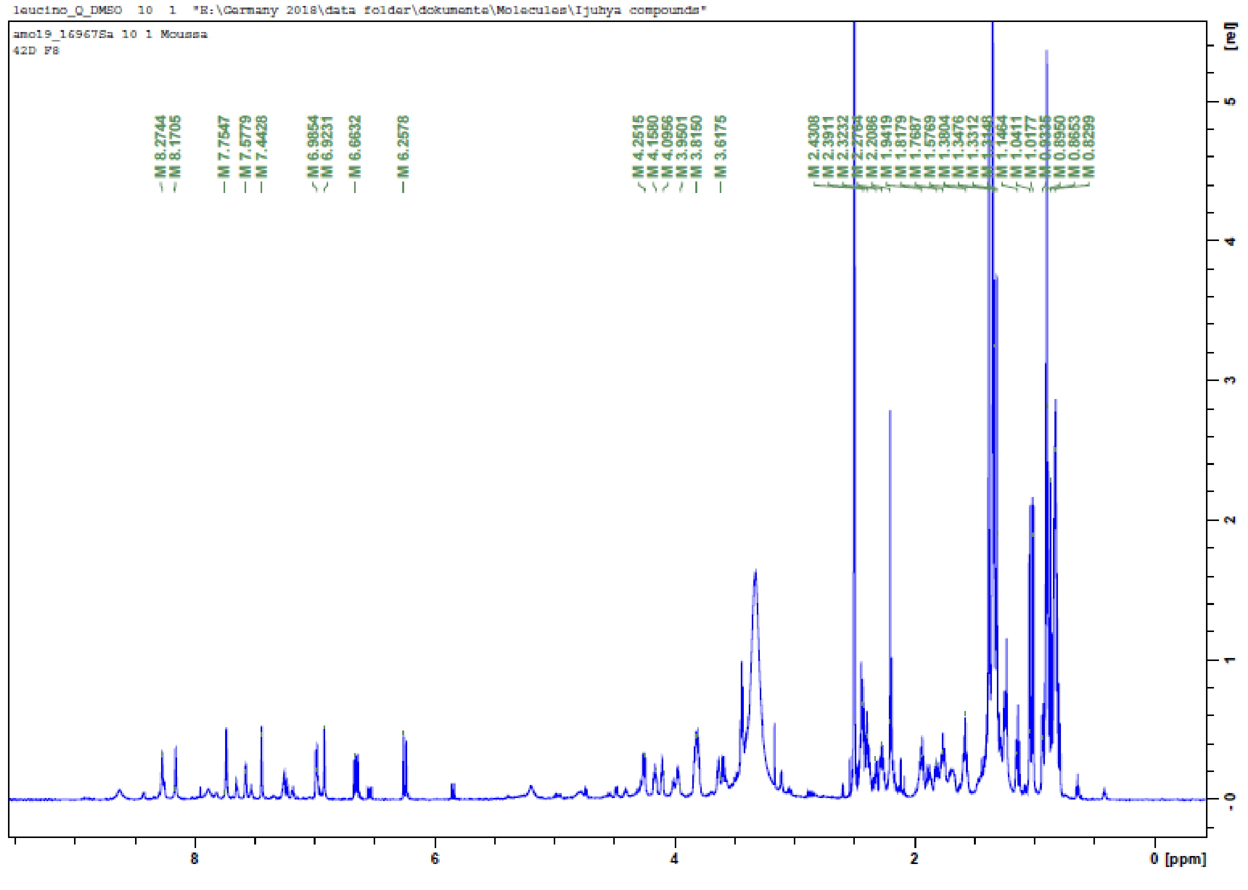

S11 figure: ^{1}HNMR spectra of **leucinostatin Q 2**

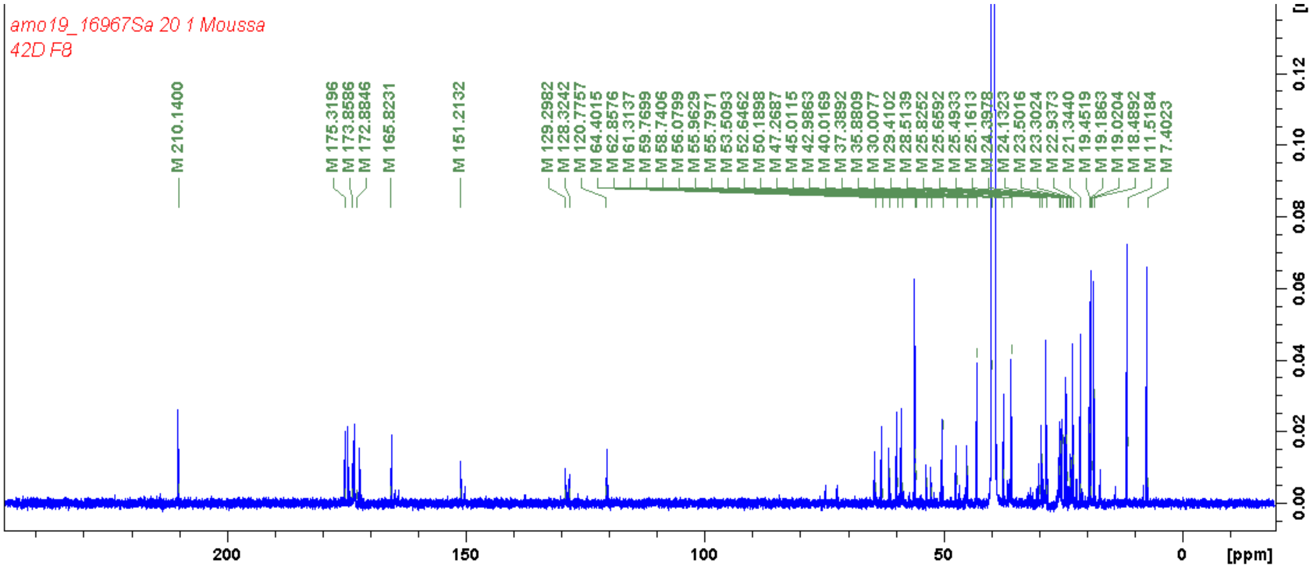

S12. Figure: ^{13}CNMR spectra of **leucinostatin Q 2.**.

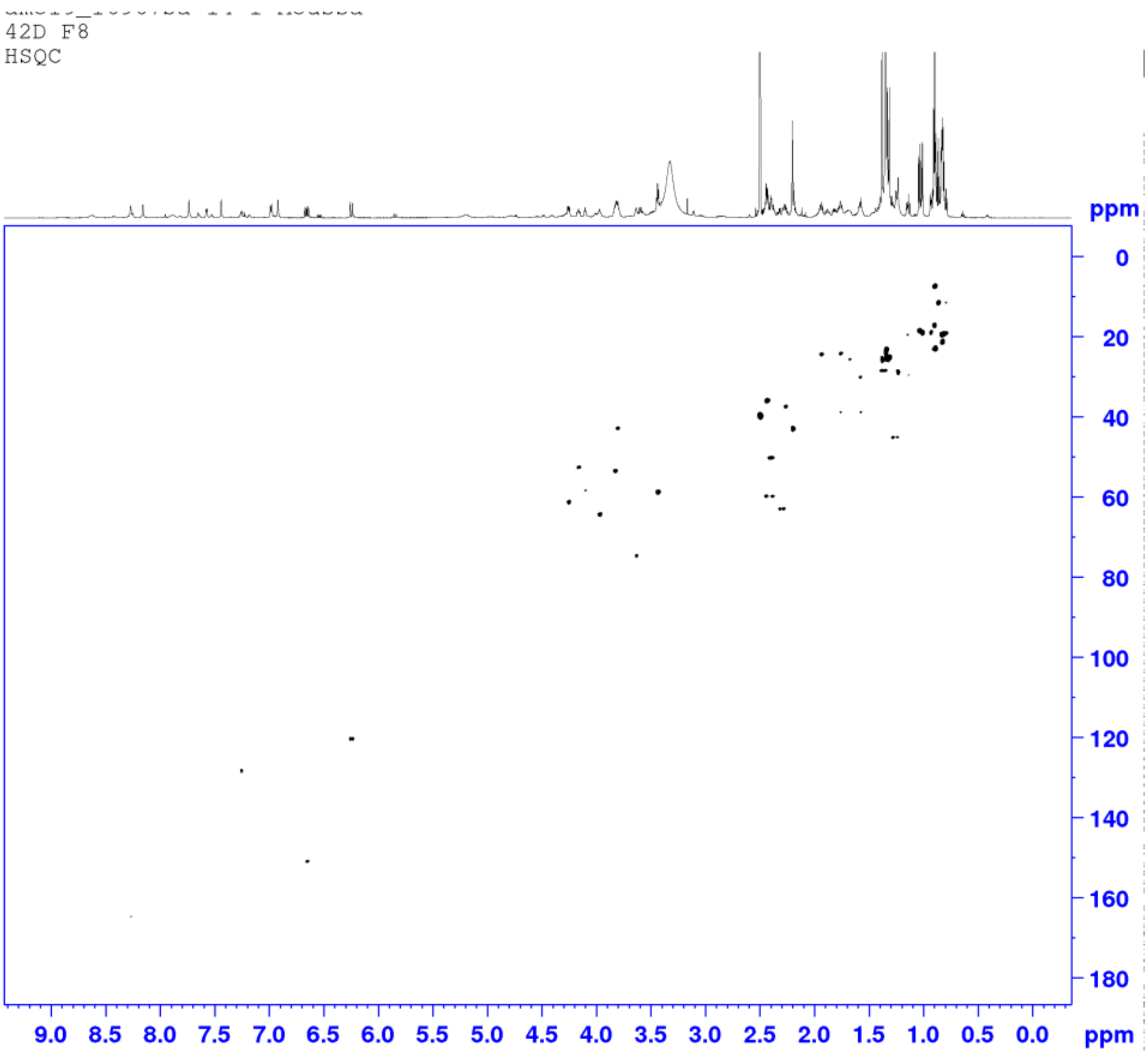
42D F8
HSQC
ppm
0
20
40
60
80
100
120
140
160
180
9.0 8.5 8.0 7.5 7.0 6.5 6.0 5.5 5.0 4.5 4.0 3.5 3.0 2.5 2.0 1.5 1.0 0.5 0.0
ppm

S13 Figure: HSQC spectra of **leucinostatin Q 2**

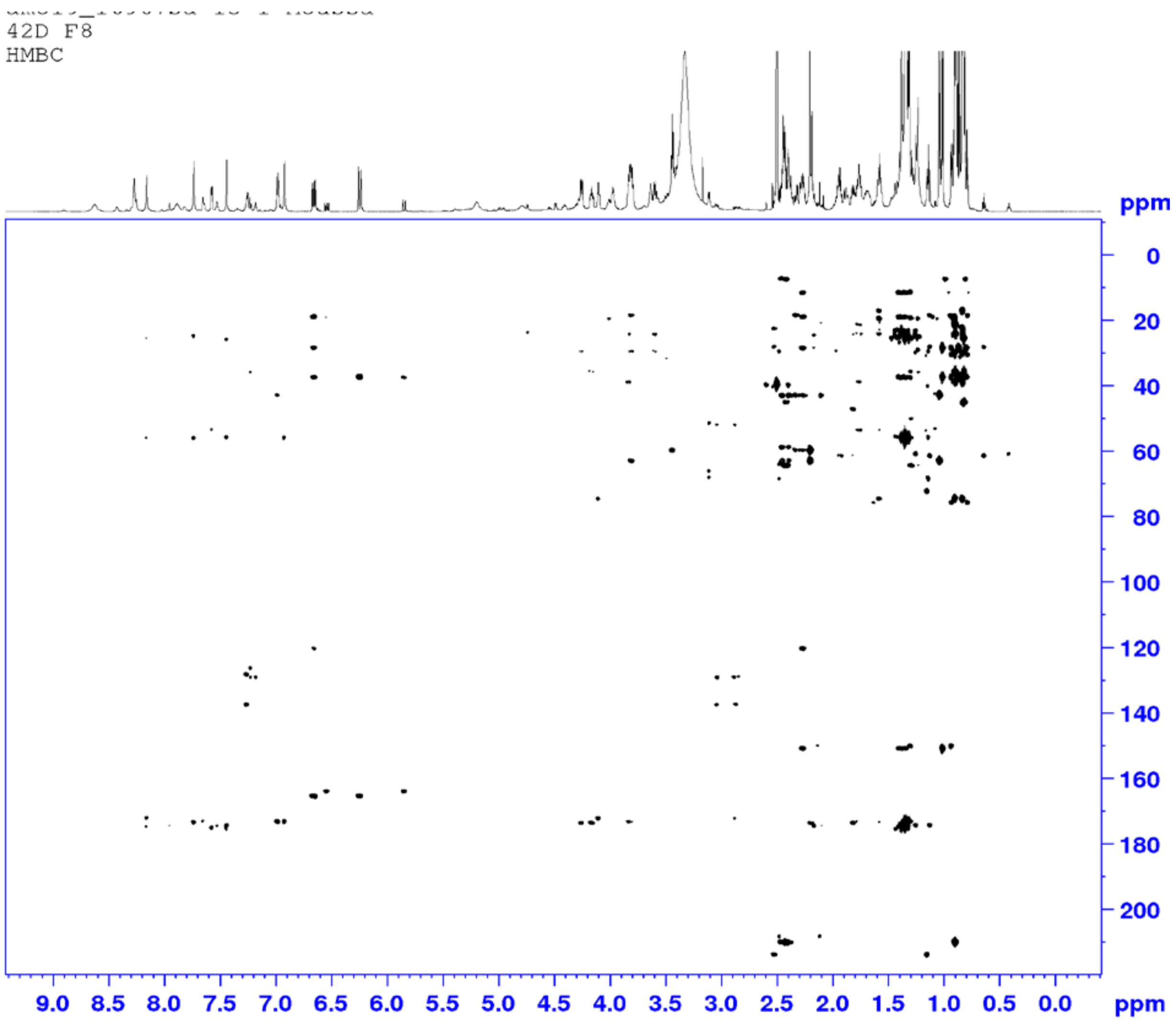

S14 Figure: HMBC spectra **of leucinostatin Q 2**

COSY F8

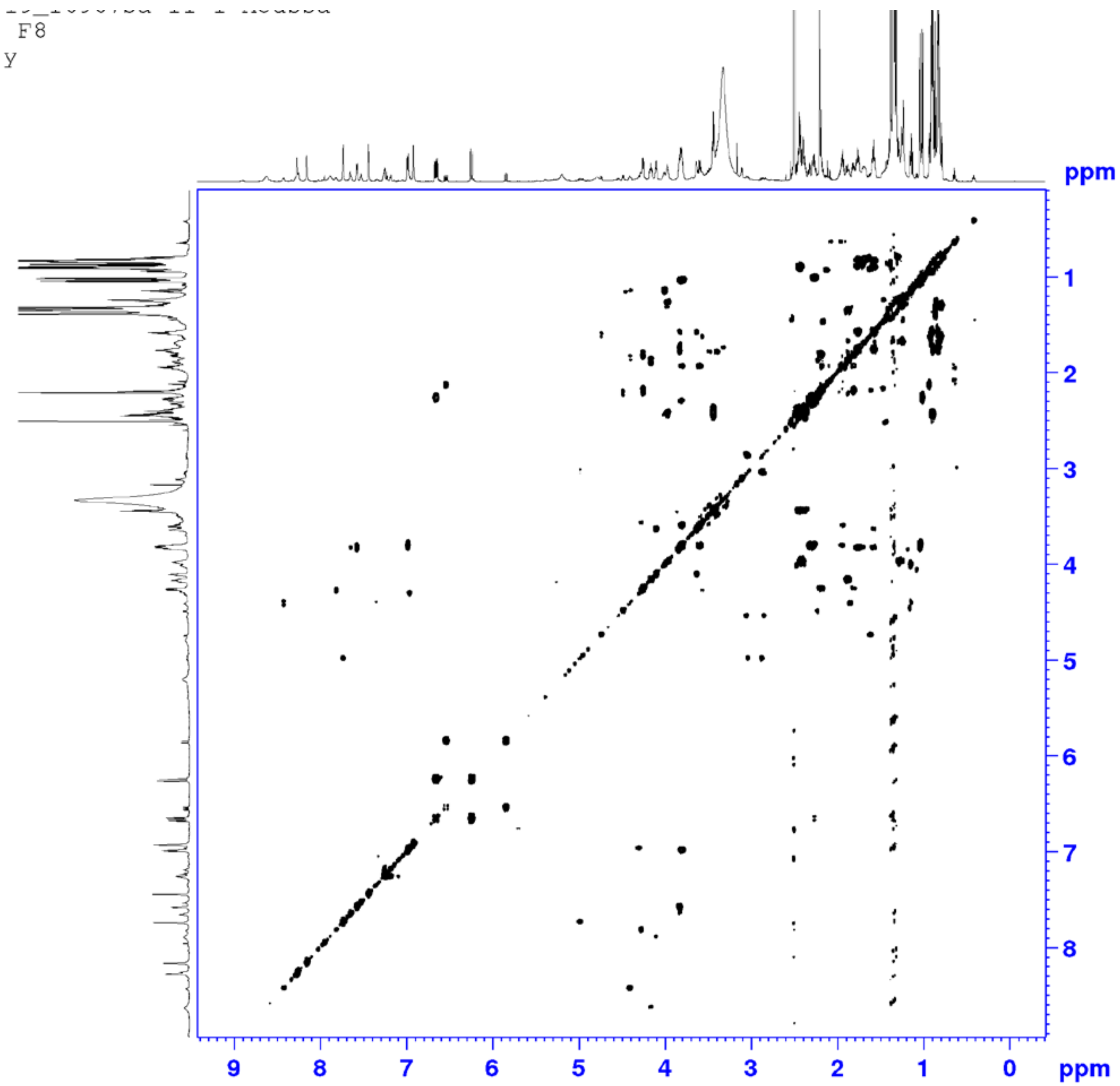

S15 Figure: COSY spectra **of leucinostatin Q 2**

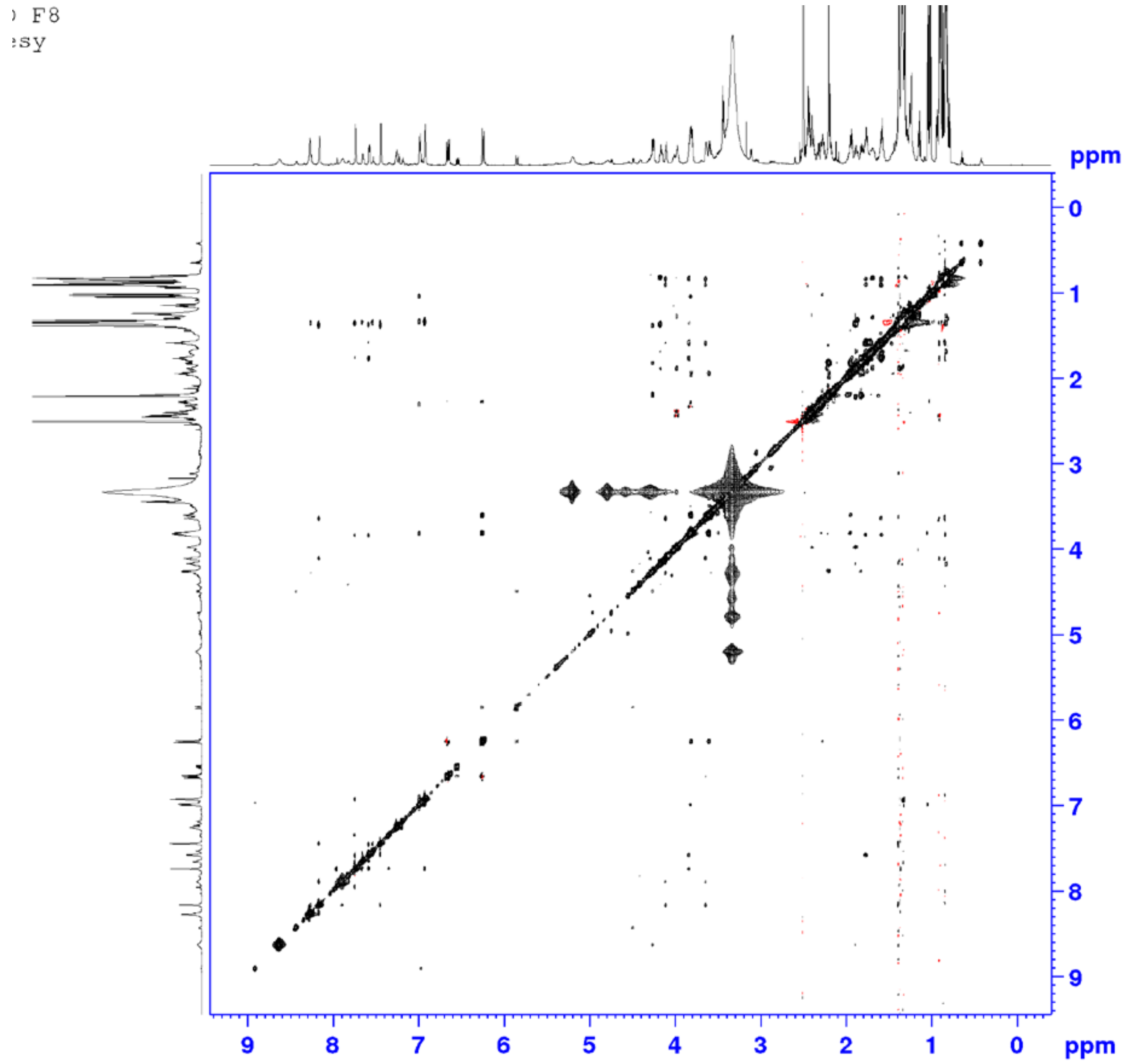

S16 Figure: NOESY spectra **of leucinostatin Q 2**

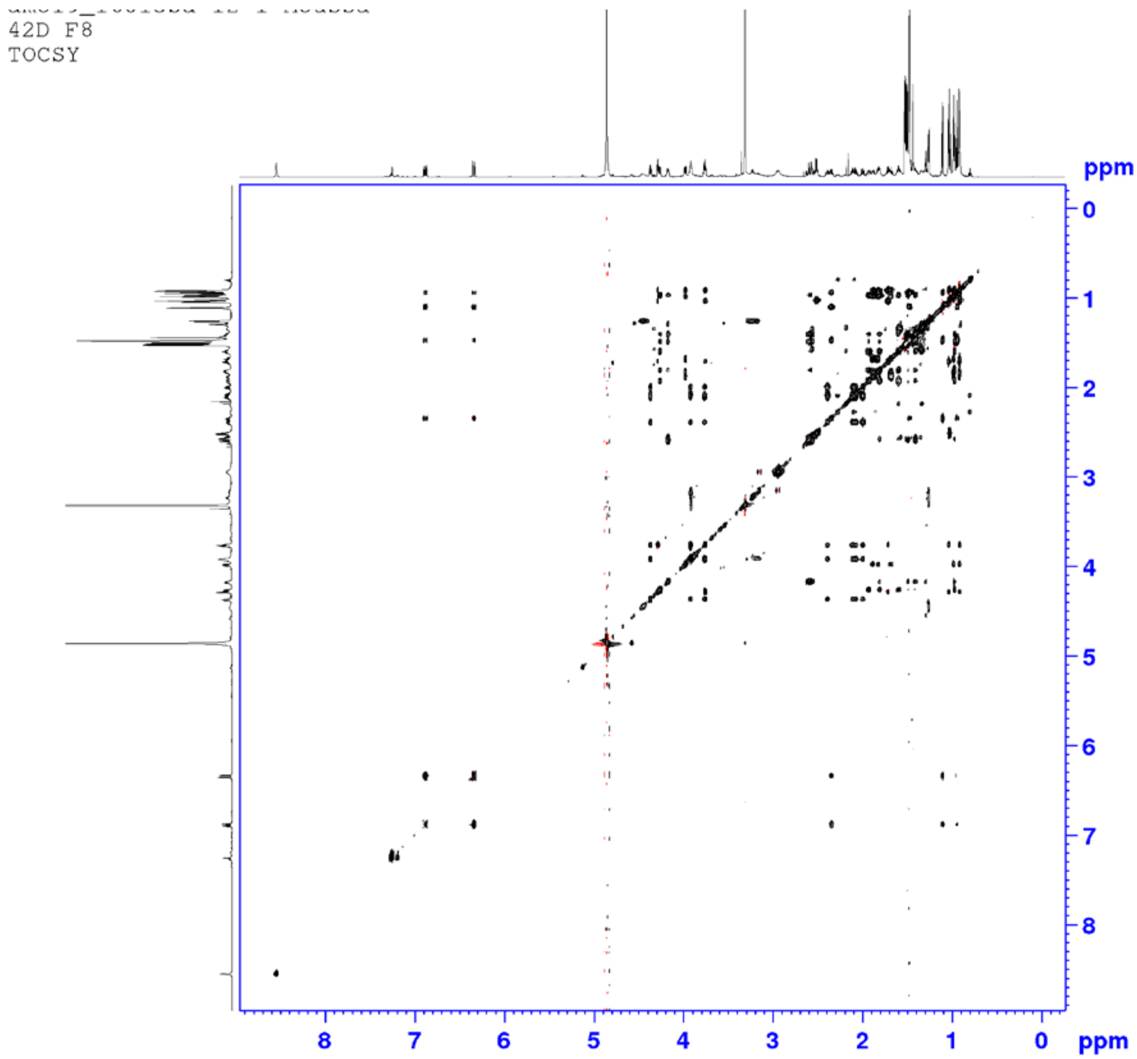
42D F8
TOCSY
ppm
0
1
2
3
4
5
6
7
8
8
7
6
5
4
3
2
1
0
ppm

S17 Figure: TOCSY spectra **of leucinostatin Q 2**

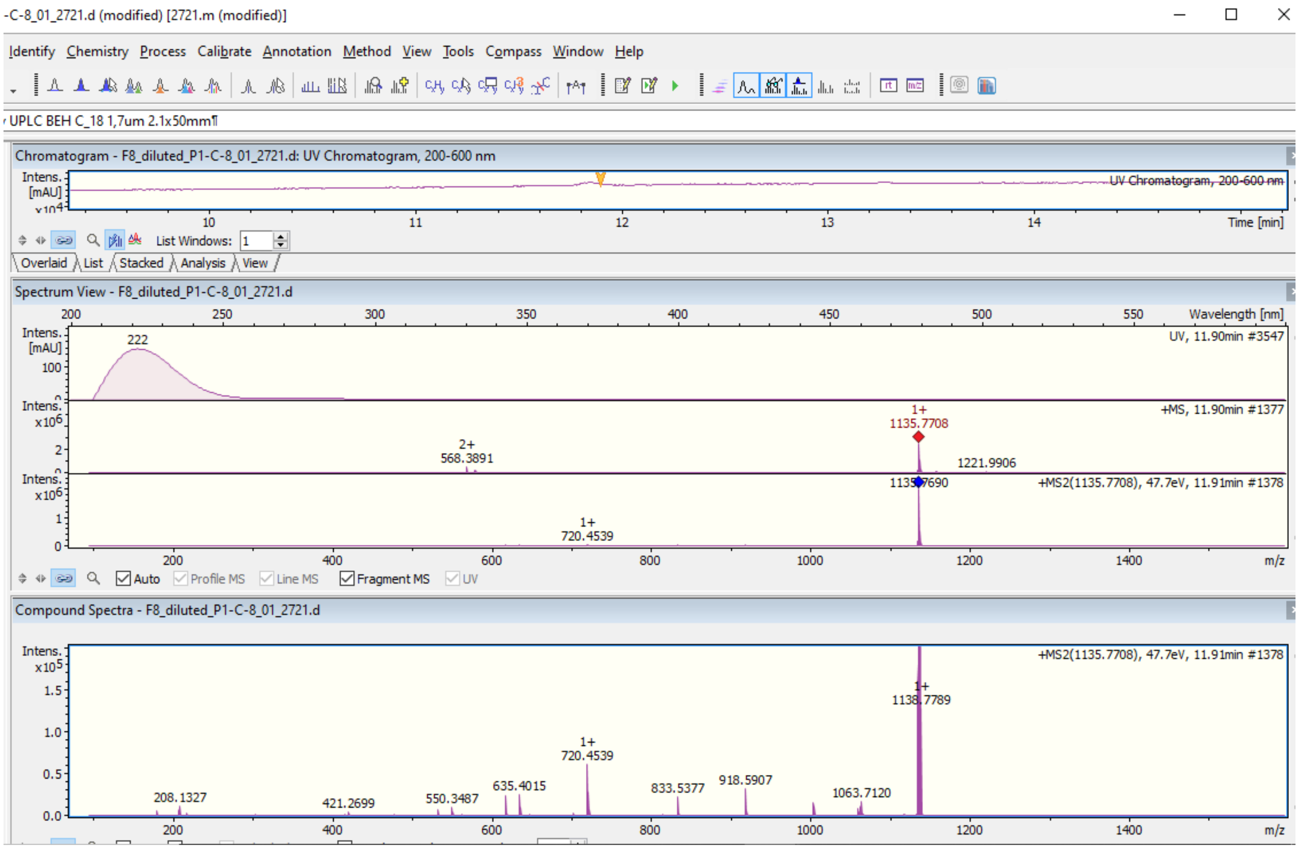

S18 Figure: HR-ESI-MS-MS fragmentation pattern of **leucinostatin Q 2.**

S3 Table. ¹H NMR and ¹³C NMR data of **leucinostatin P 3** and its characteristic chemical shift values.

residue	position	δC, type	δH(J in Hz)	residue	position	δC, type	δH(J in Hz)
**ΔMHA**	**1**	165.28,C		**Aib2**	**1**	173.23,C	
	**2**	120.19,CH	6.22		**2**	55.8,C	
	**3**	150.59,CH	6.64		**3**	25.18,CH3	1.39
	**4**	37.17,CH	2.25		**4**	23.06,CH3	1.33
	**5**	28.22,CH2	1.36		**NH**		7.47
	**6**	11.29,CH3	0.87	**Leu**	**1**	173.78,C	
	**7**	18.62,CH3	1.01		**2**	53.88,CH	3.79
**Pro**	**1**	173.16,C			**3**	38.74,CH2	1.55
	**2**	61.28,CH	4.24				1.75
	**3**	29.19,CH2	1.81		**4**	23.79,CH	1.77
			2.19		**5**	21.27,CH3	0.85
	**4**	24.10,CH2	1.95		**6**	22.58,CH3	0.91
	**5**	46.90,CH2	3.82		NH		7.02
			3.56	**Aib3**	**1**	175.23,C	
**AHMOD**	**1**	173.63,C			**2**	24.30,CH3	1.34
	**2**	52.65,CH	4.15		**3**	25.01,CH3	1.33
	**3**				**4**	56.5,C	
					**NH**		7.6
	**4**	25.11,CH	1.7	**Aib4**	**1**	171.4,C	
	**5**	44.90,CH2	1.21		**2**	55.7,C	
			1.28		**3**	23.85,CH3	1.35
	**6**	64.02,CH	3.96		**4**	24.10,CH3	1.32
	**7**	51.12,CH2	2.57		**NH**		7.6
			2.54	**C-terminal**	**1**	43.62,CH	3.85

	**8**	209.95,C		**2**	54.13,CH2	2.5
	**9**	35.66,CH2	2.43			2.48
	**10**	7.20,CH3	0.9	**3**	51.12,CH2	2.57
	**11**	22.58,CH3	0.91			2.54
	**NH**		6.93	**4**	60.18,CH2	3.4
**Hyleu**	**1**	172.16,C		**NH**		8.25
	**2**	63.85,CH	4.01			
	**3**	73.88,CH	3.67			
	**4**	29.57,CH	1.58			
	**5**	13.50,CH3	0.85			
	**6**	18.98,CH3	0.81			
	**NH**		7.8			
**Aib1**	**1**	174.68,C				
	**2**	56.03,C				
	**3**	23.57,CH3	1.33			
	**4**	26.01,CH3	1.36			
	**NH**					
		8.43				

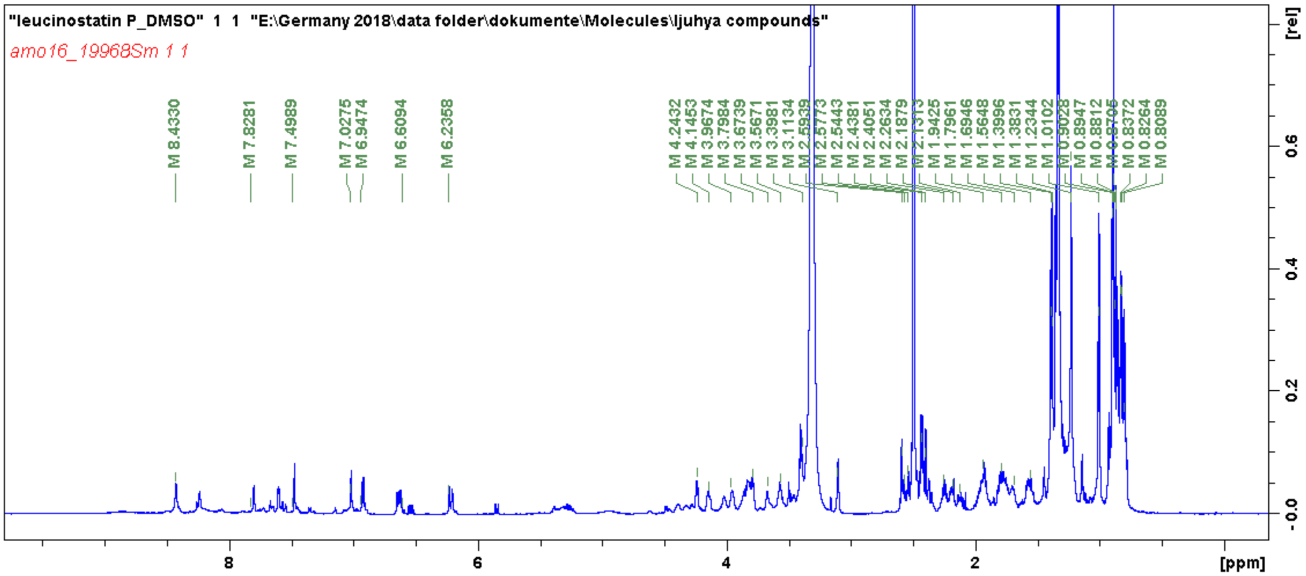

S19 figure: 1HNMR spectra of **leucinostatin P 3**

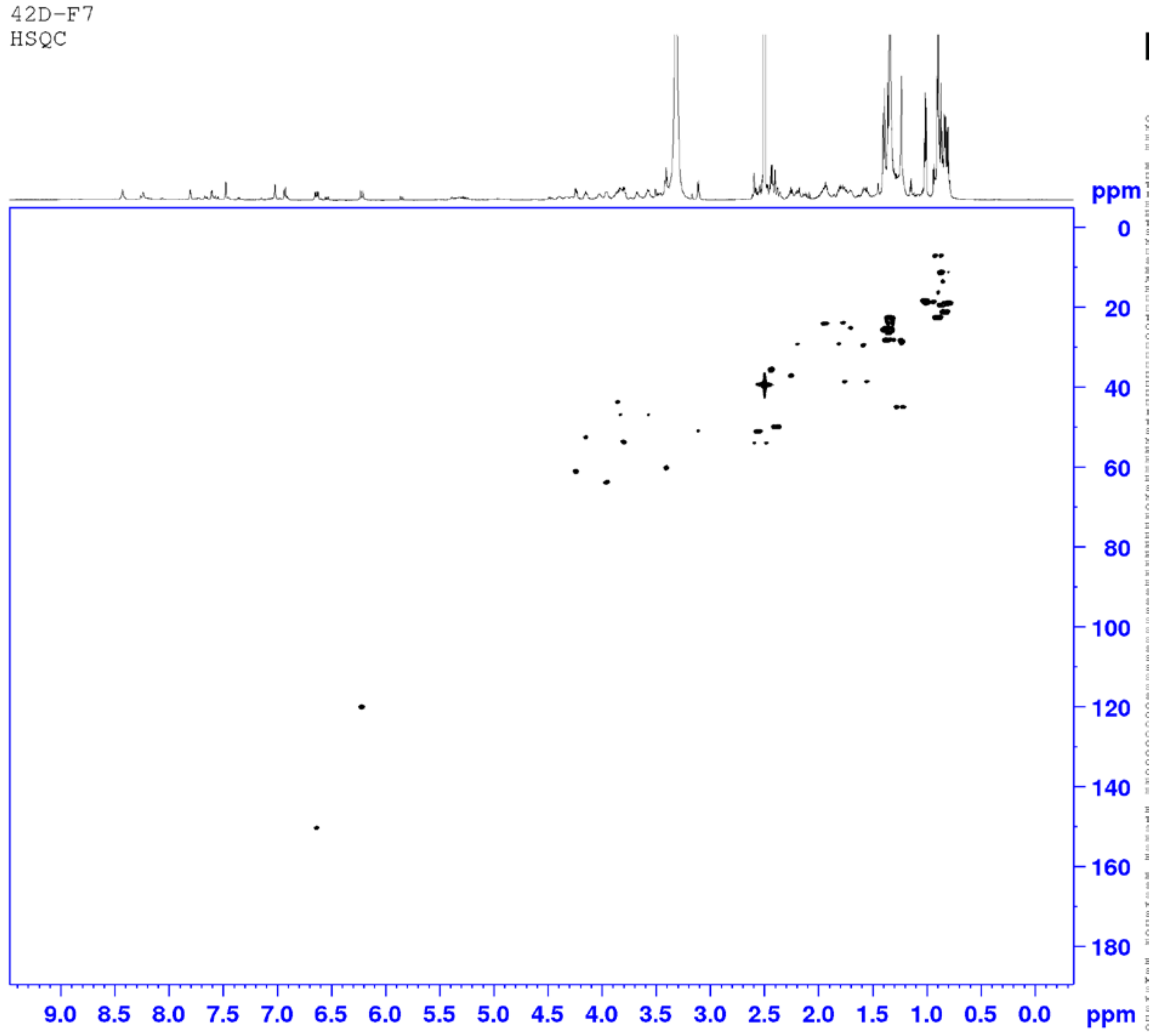

S20 Figure: HSQC spectra of **leucinostatin P 3**

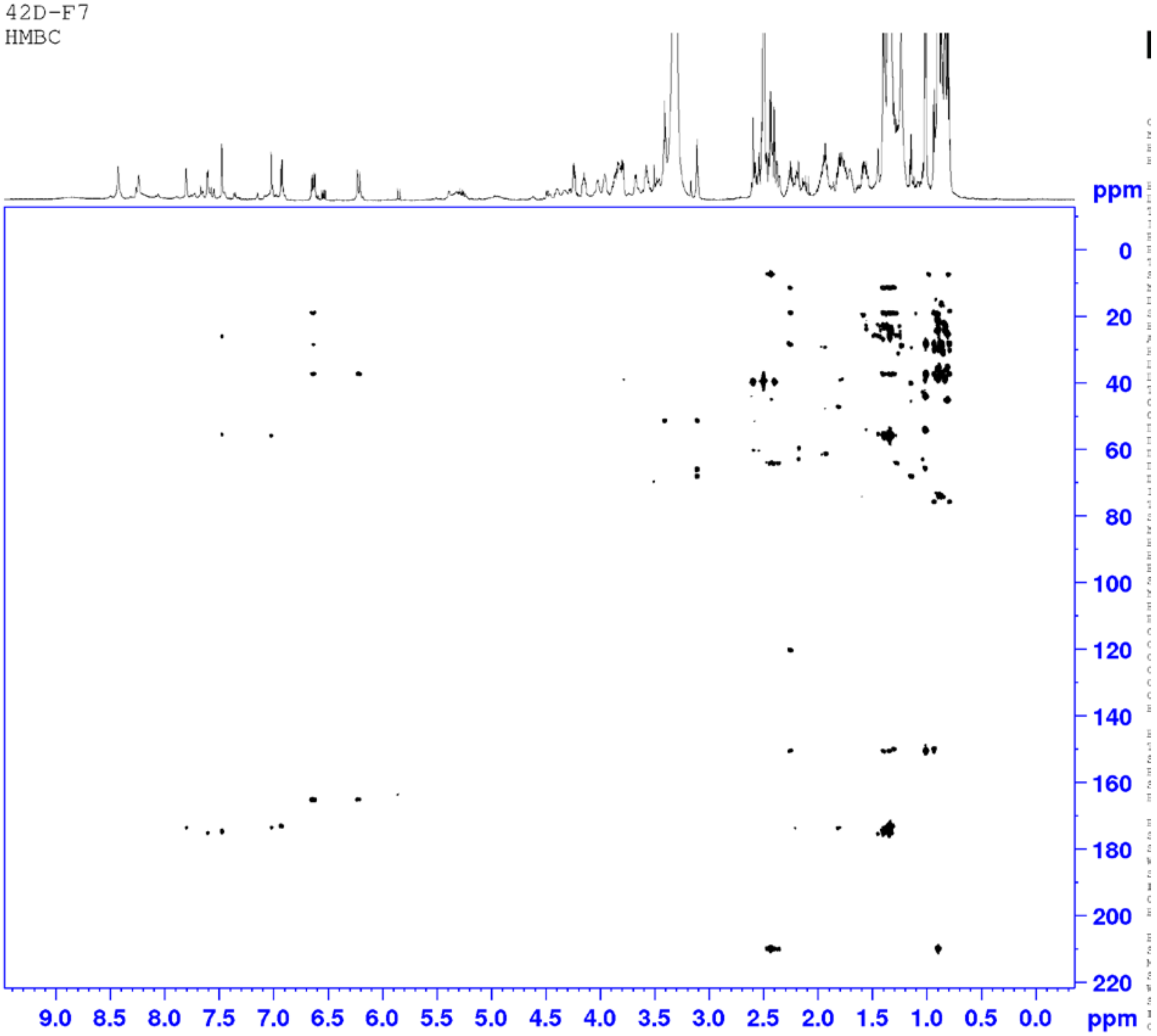

S21 Figure: HMBC spectra **of leucinostatin P 3**

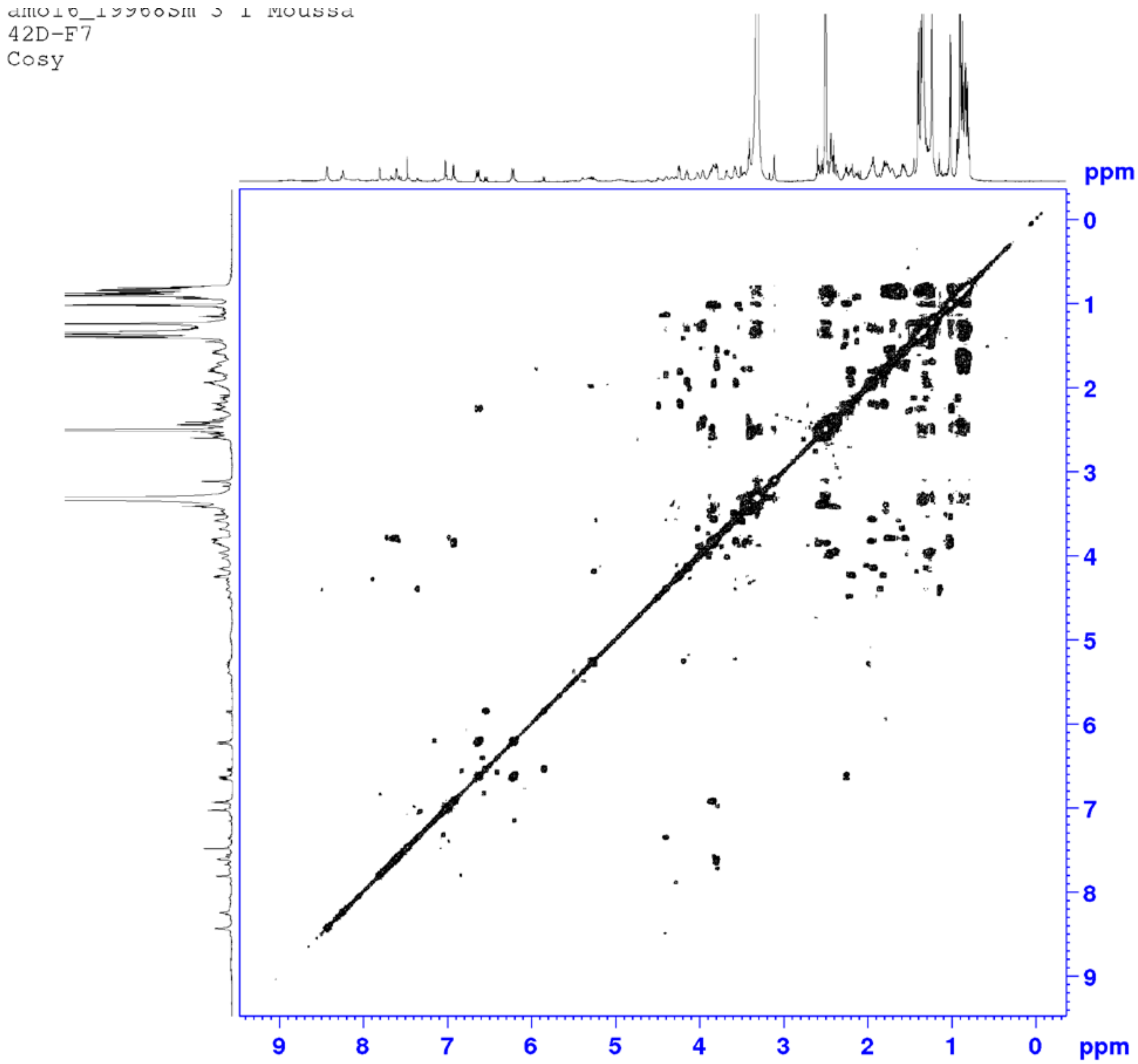

S22 Figure: COSY spectra **of leucinostatin P 3.**

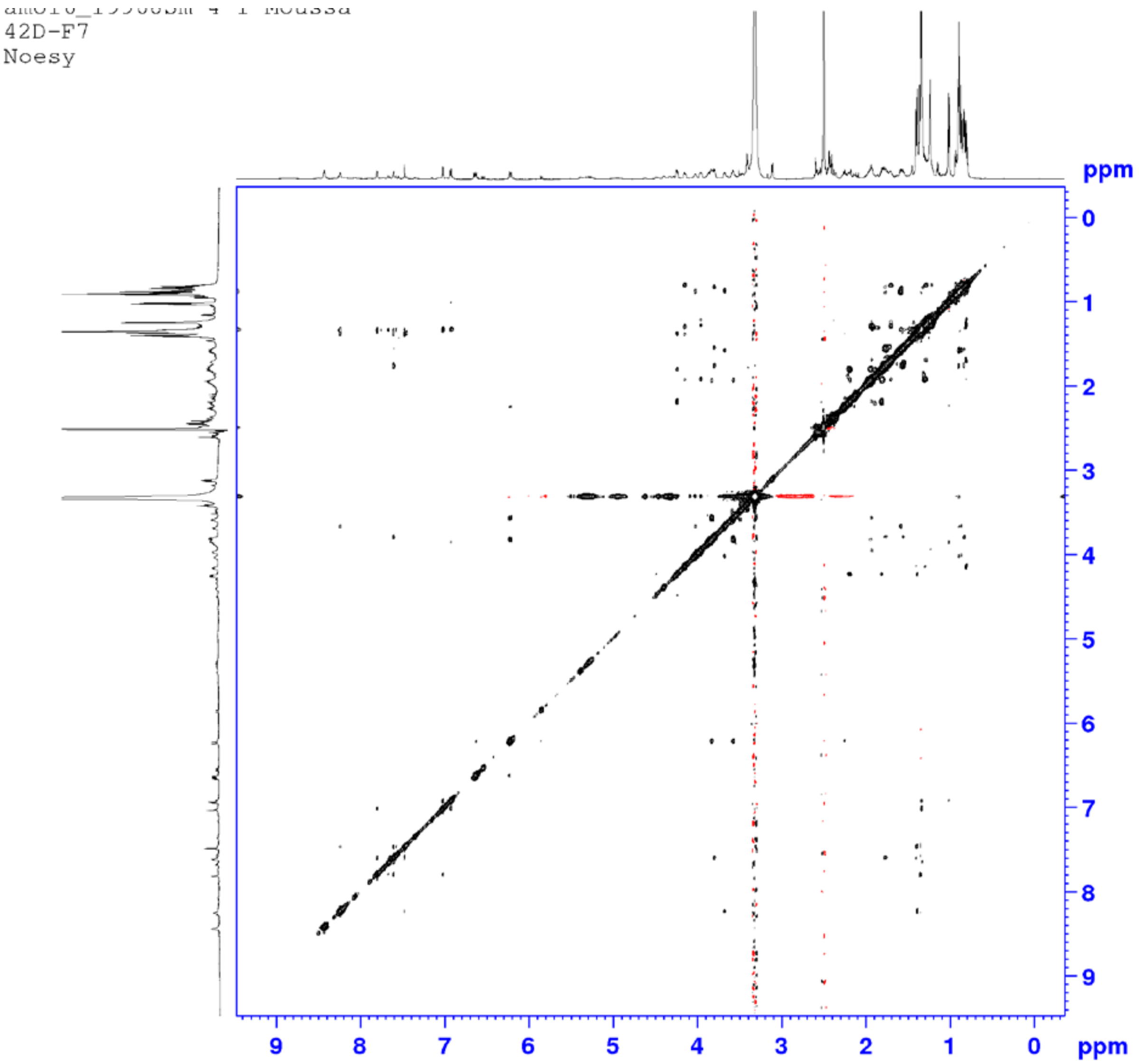

S23 Figure: NOESY spectra **of leucinostatin P 3**

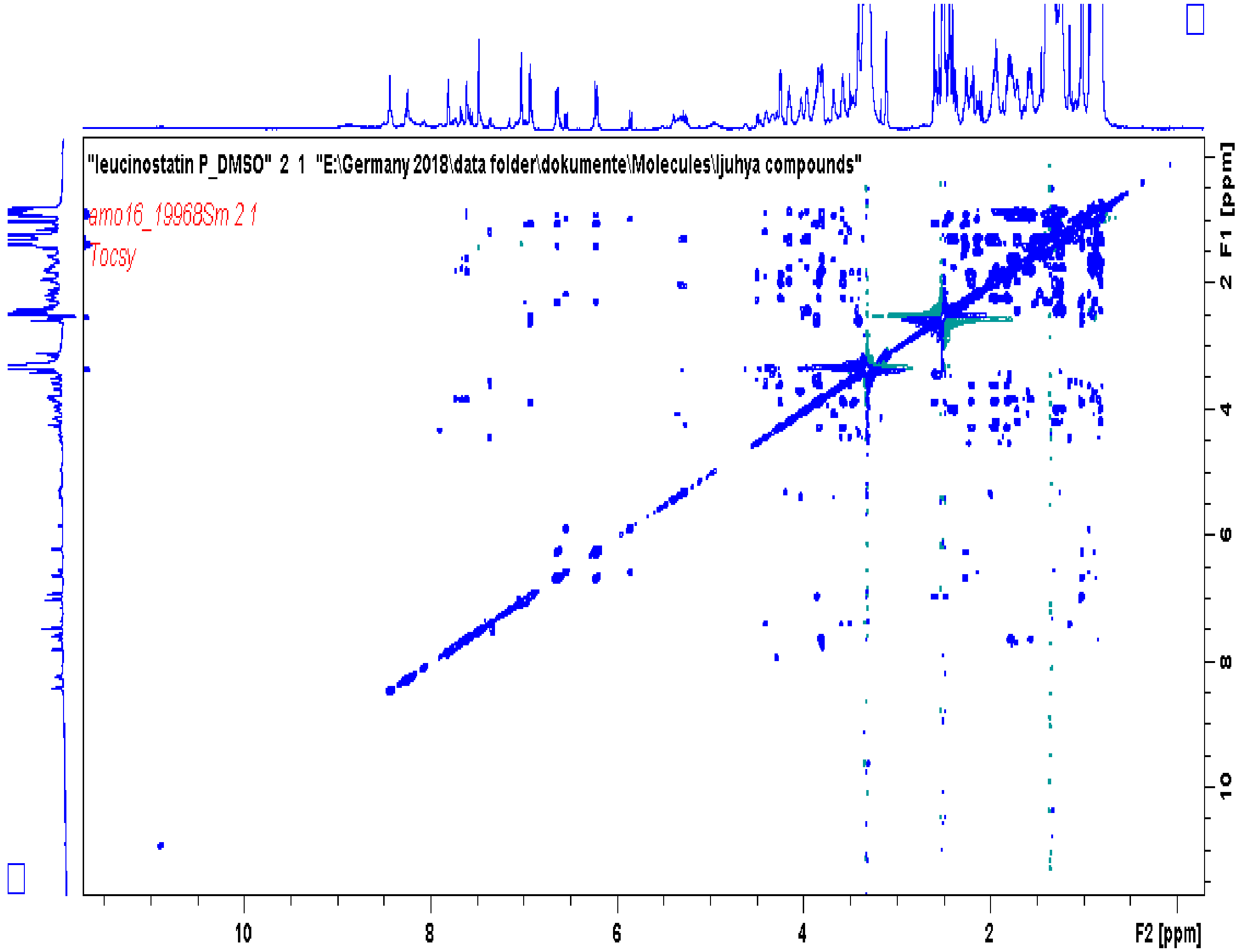

S24 Figure: TOCSY spectra **of leucinostatin P 3.**

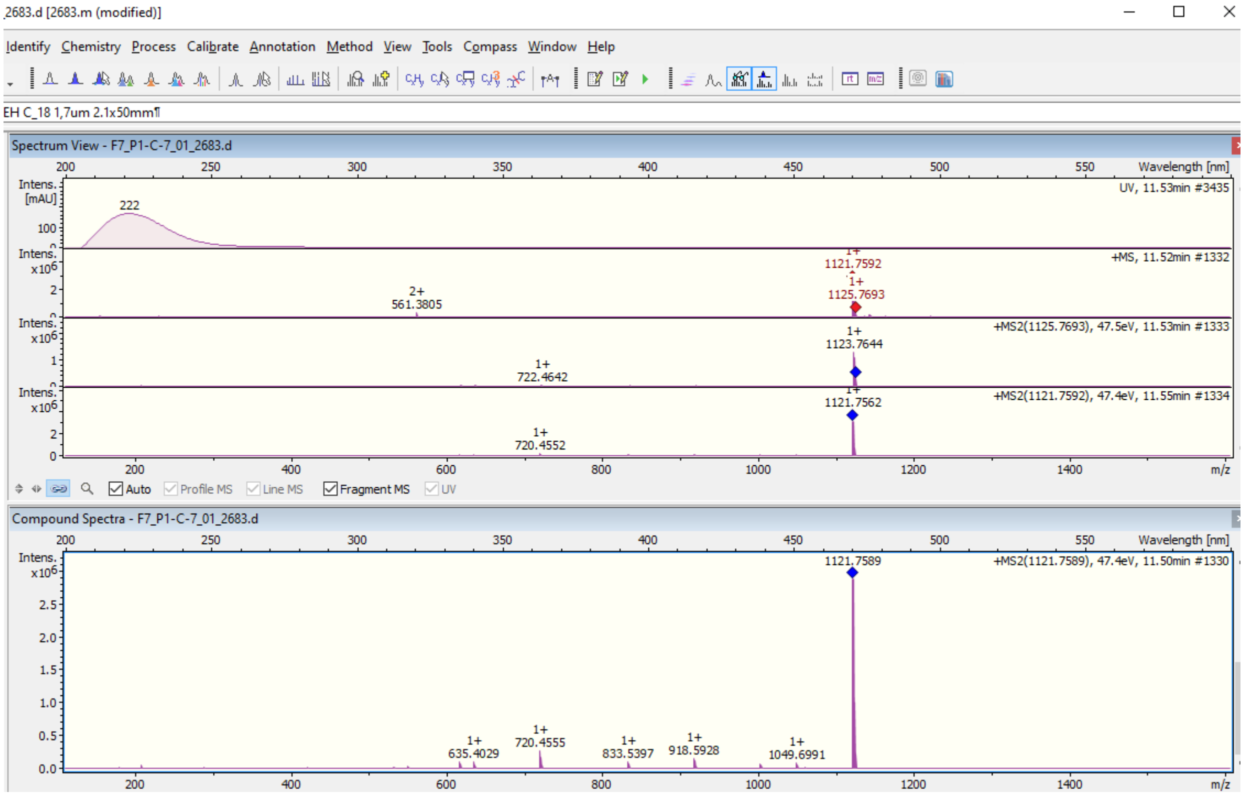

S25 Figure: HR-ESI-MSMS fragmentation pattern of **leucinostatin P 3.**

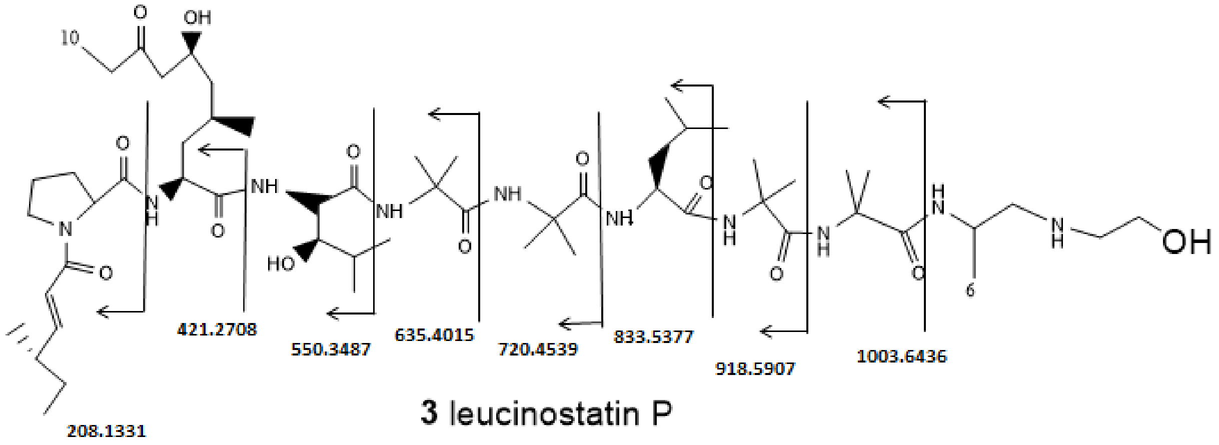

3 leucinostatin P

S26 Figure: HR-ESI-MS-MS fragments of **leucinostatin P 3.**

S4 Table. ^{1}H NMR and ^{13}C NMR data of **arthrichitin D 4** and its characteristic chemical shift values

			Arthrichitin D			
**residue**	**position**	**δC, type**	**δH(J in Hz)**		**δH(J in Hz)**	**δC, type**
**L-Glu**	**1**	169.92,C			**Dimethyl-oxy-hexadecanoyl**	
	**2**	52.50,CH	4.19	**27**	173.19,C	
	**3**	25.20,CH2	1.82	**28**	40.70,CH	3.11
			2.04	**29**	82.30,CH	4.91
	**4**	30.50,CH2	2.25	**30**	130.20,C	
			2.4	**31**	129.9,CH	6.12
	**5**	173.02,C		**32**	125.70,CH	6.33
	**NH**		7.35	**33**	136.50,CH	5.84
**L-Gly**	**6**	169.30,C	4.05	**34**	32.10,CH2	2.19
	**7**	43.40,CH2	3.73	**35**	31.50,CH2	1.34
			3.9	**36**	28.9,CH2	1.35
	**NH**		8.06	**37**	24.40,CH2	1.33
**L-Phe**	**8**	171.50,C		**38-40**	22.10,CH2	1.36
	**9**	55.01,CH	4.13	**42**	14.74,CH3	0.9
	**10**	36.60,CH2	3.43	**43**	14.04,CH3	0.96
				**44**	11.25,CH3	1.64
	**11**	138.55,C				
	**12**	129.40,CH	7.34			
	**13**	128.34,CH	7.34			
	**14**	126.25,CH	7.26			
	**15**	128.22,CH	7.38			
	**16**	129.30,CH	7.27			
	**NH**		8.81			
**L-Gln**	**17**	171.60,C				
	**18**	56.60,CH	3.83			
	**19**	26.00,CH2	1.71			
	**20**	32.37,CH2	2.18			
	**21**	170.13,C				
	**NH**		7.97			
**L-Val**						
	**22**	170.0,C				
	**23**	55.6,CH	4.56			
	**24**	33.27,CH	2.04			
	**25**	19.17,CH3	0.89			
	**26**	17.79,CH3	0.88			
	**NH**		7.98			

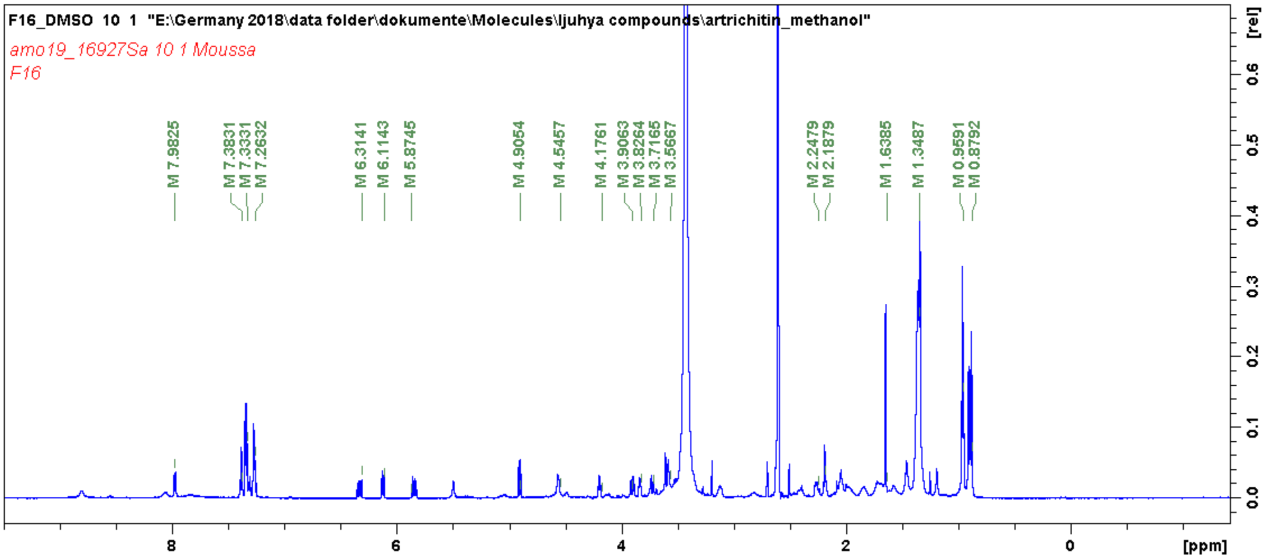

S27.Figure: ^{1}H NMR spectra of **arthrichitin D 4**

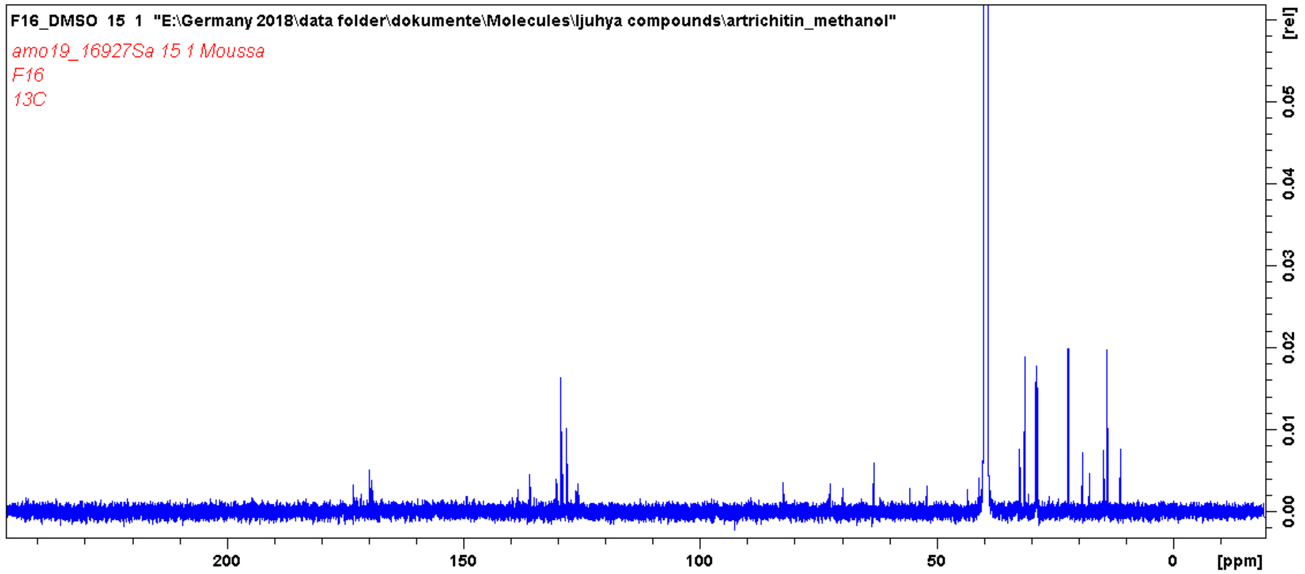

S28 Figure: ^{13}CNMR spectra of **arthrichitin D 4**

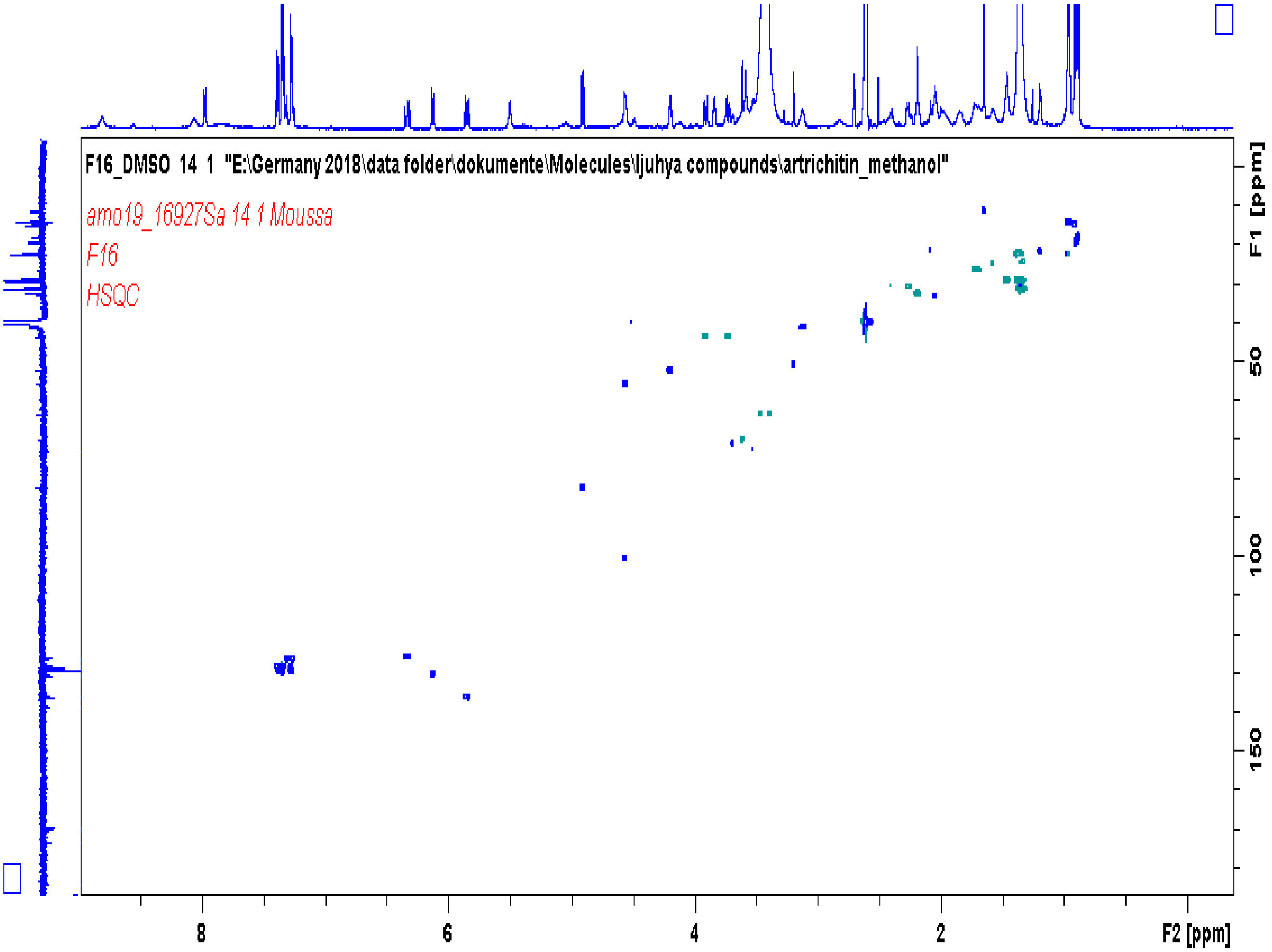

S29 Figure: HSQC spectra of **arthrichitin D 4**

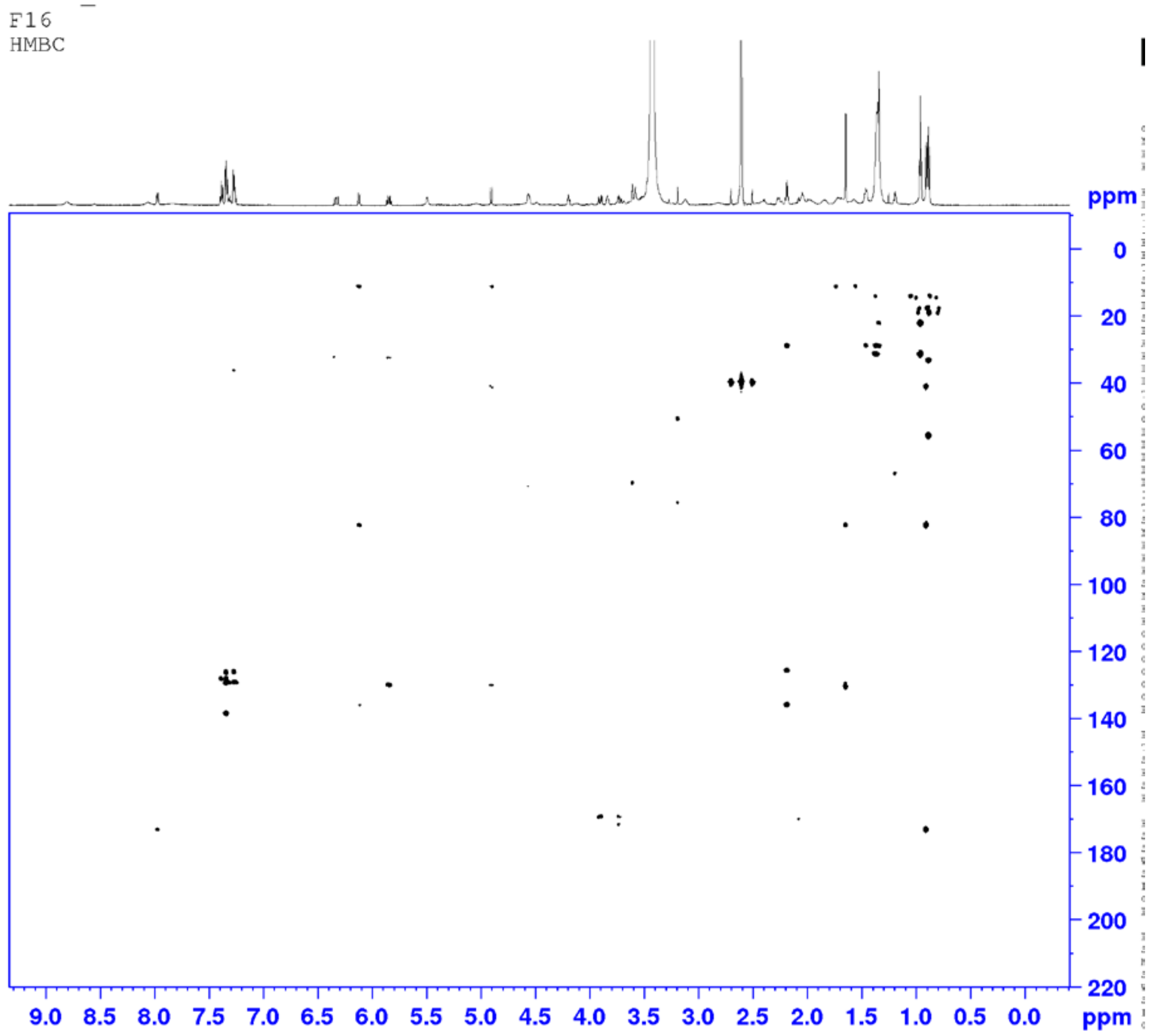

S30 Figure: HMBC spectra **of arthrichitin D 4..**

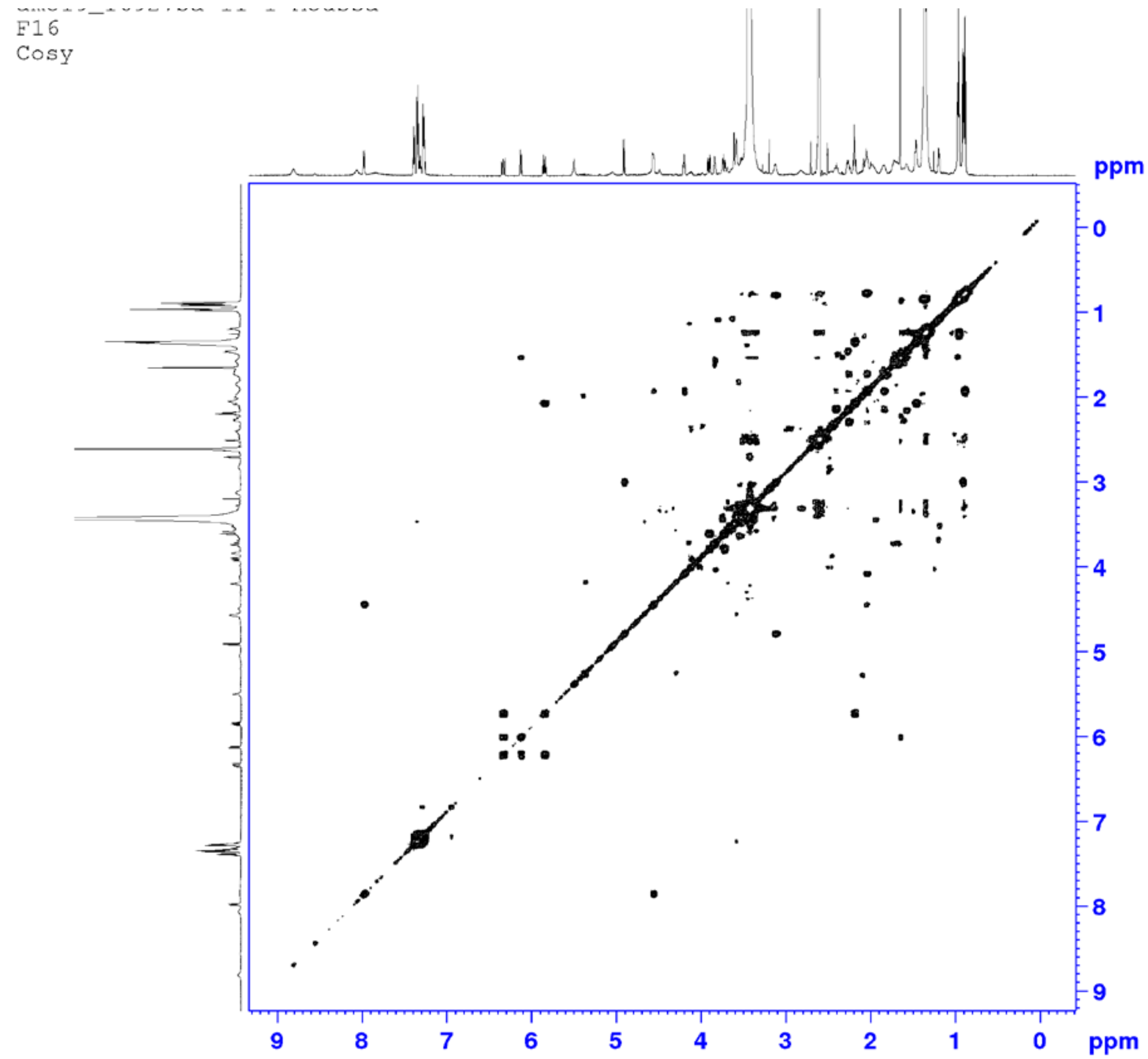

S31 Figure: COSY spectra **of arthrichitin D 4**

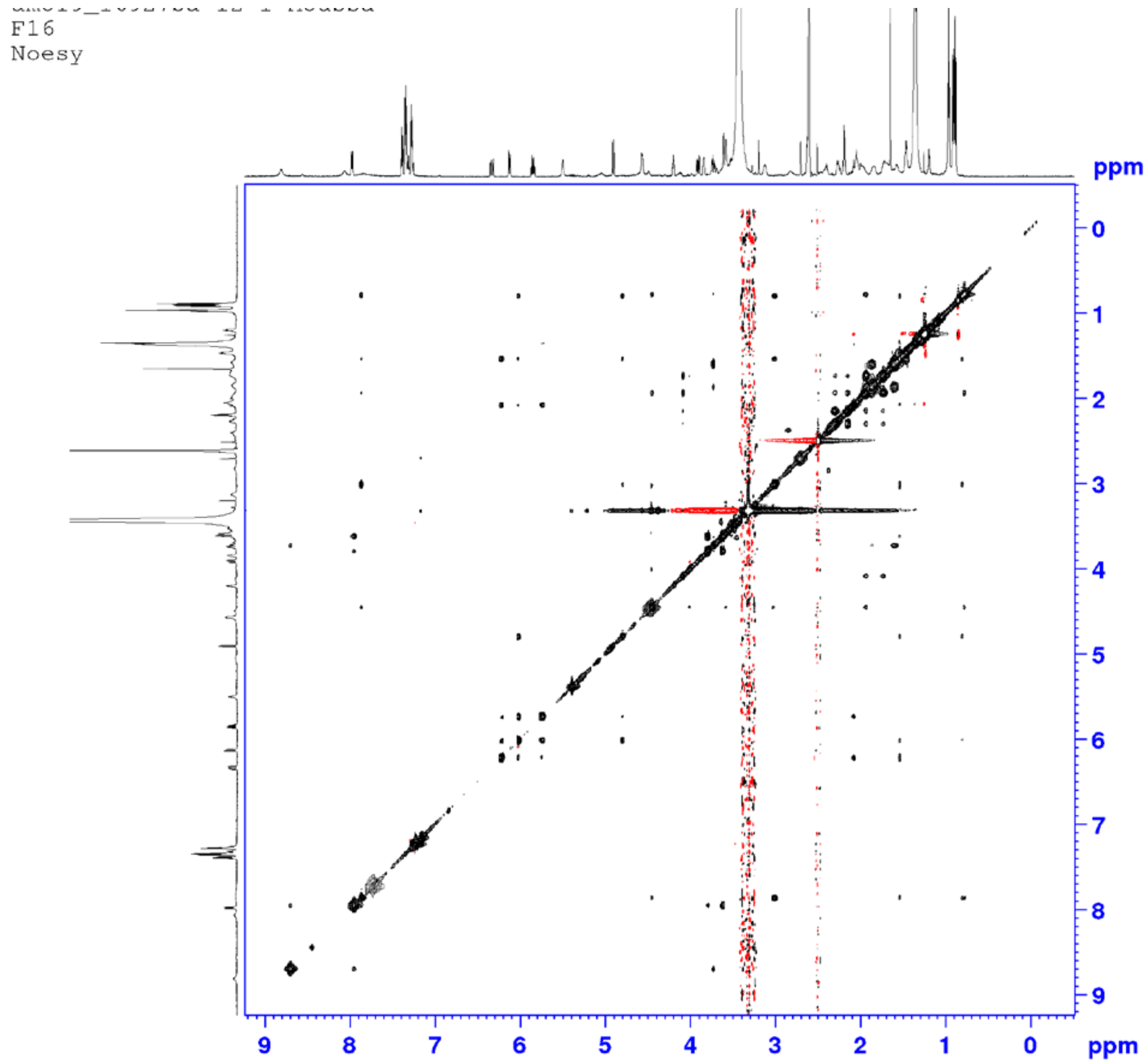

S32 Figure: NOESY spectra **of arthrichitin D 4**

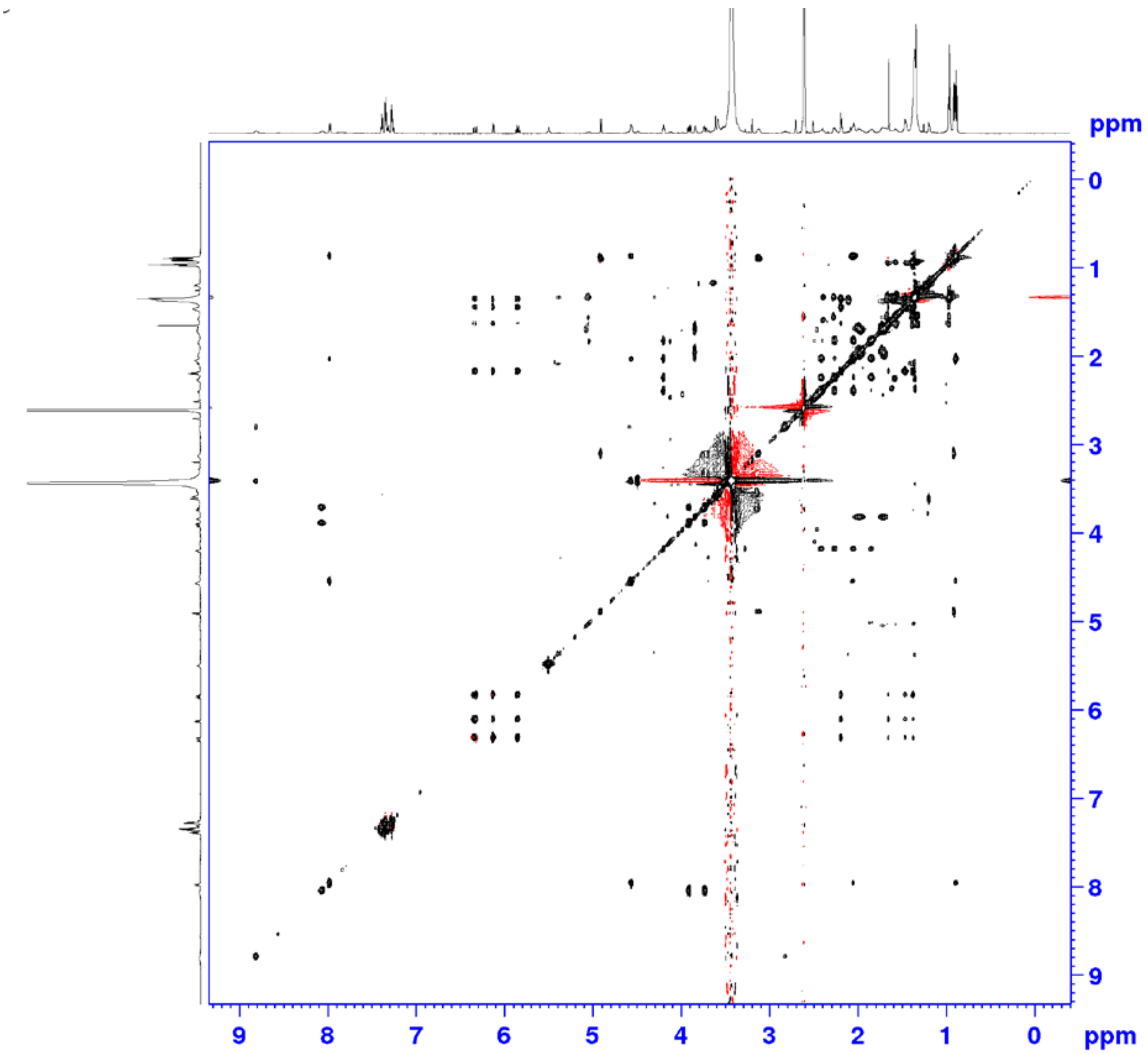

S33 Figure: ROESY spectra **of arthrichitin D 4**

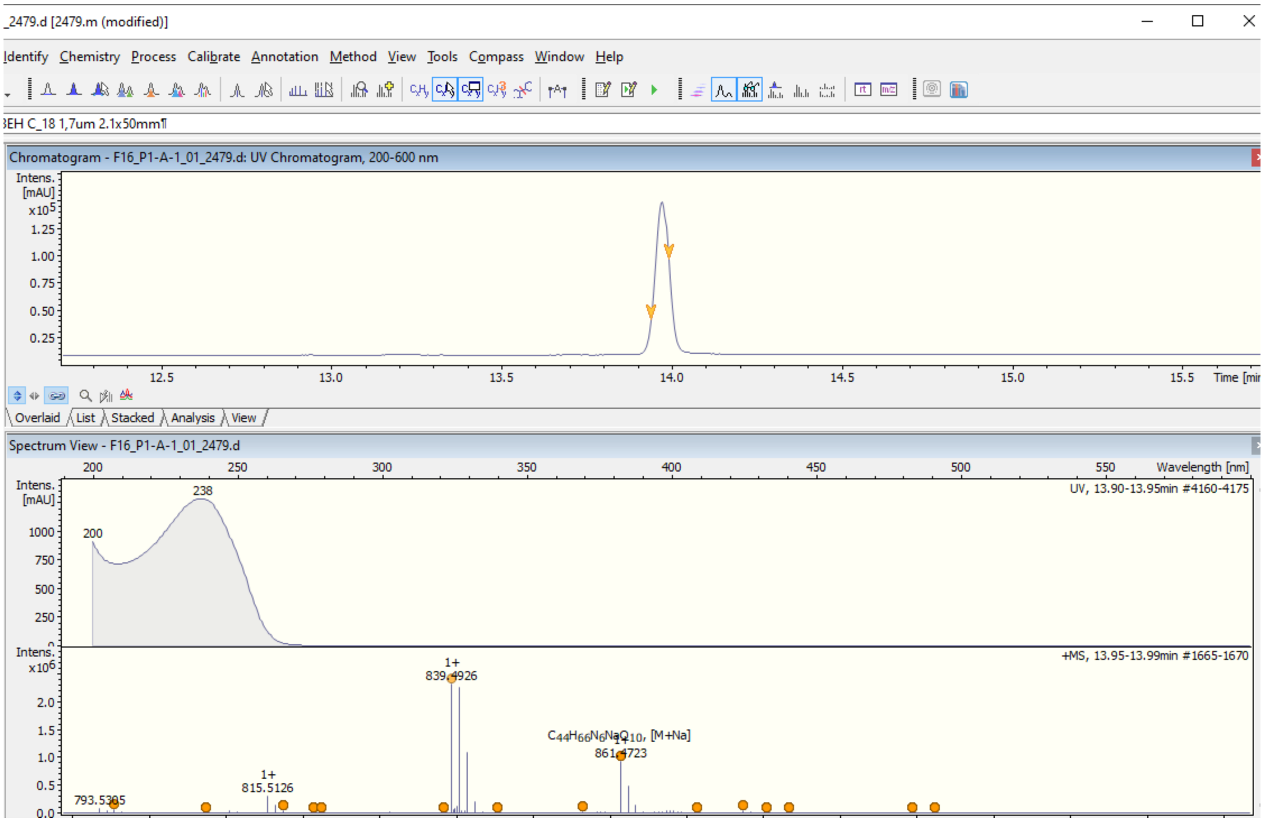

S34.Figure: HR-ESI-MS of **arthrichitin D 4.**

S12 Text: ^{1}H NMR and ^{13}C NMR data of **chaetoglobosin B 5** and its characteristic chemical shift values.

Chaetoglobosin B 5

Yellow powder, UV absorption λ_{max} 200, 222, 281 nm , HR-ESI-MS m/z 1057.5321 $[2M+H]^+$ (calcd for $C_{32}H_{36}N_2O_5$); ^{1}H NMR (700 MHz, methanol-d4) δH 3.53 (H-3, dd, J=8.4, 5.2 Hz), 3.84 (H-7, d, J= 9.4 Hz), 2.07 (H-8, dd, J= 9.4, 12.3 Hz), 2.87 (H-10a, dd, J=5.3, 14.5 Hz), 2.57 (H-10b, dd, J=5.3, 14.5 Hz), 1.28 (H-11, s), 1.65 (H-12, s), 6.05 (H-13, dd, J=9.4, 15.9 Hz), 5.15 (H-14, ddd, J=3.5, 10.2, 15.5 Hz), 2.32(H-15a, m), 2.01 (H-15b,m), 2.58 (H-16, m), 5.57(H-17, d, J=9.6 Hz), 5.11 (H-19,s), 6.68 (H-21, d, J=16.2 Hz), 7.46 (H-22,d J=5.6 Hz), 7.02 (H-2′, brs), 7.48 (H-4′, m), 7.01 (H-5′, t, J=7.1 Hz), 7.07(H-6′, t, J= 7.3 Hz), 7.32 (H-7′, d, J= 8.3Hz), 1.01(Me-16, d, J=6.8 Hz), 1.42 (Me-18, s).

S13 Text: ^{1}H NMR and ^{13}C NMR data of **19-O-acetyl chaetoglobosin B (6)** and its characteristic chemical shift values.

19-O-acetyl chaetoglobosin B 6

Yellow powder, UV absorption λ_{max} 199, 223, 280 nm, HR-ESI-MS m/z 571.2801 $[M+H]^+$, HR-ESI-MS m/z (calcd for $C_{34}H_{38}N_2O_6$); ^{1}H NMR (700 MHz, methanol-d4) δH 3.60 (H-3, dd, J=3.2,8.5 Hz), 3.26 (H-4, m), 3.89 (H-7, d, J= 9.4 Hz), 2.10 (H-8, m), 2.89 (H-10a,dd, J=14.3, 4.3 Hz), 2.60 (H-10b,dd, J=14.3, 4.3 Hz), 1.32 (H-11, s), 1.65 (H-12, s), 6.17 (H-13, dd, J=15.3, 9.5 Hz), 5.19 (H-14, ddd, J= 3.5, 9.5, 15.4 Hz), 2.01 (H-15a, m), 2.32 (H-15b, m), 2.61 (H-16, m), 5.77 (H-17, d, J=9.4 Hz), 5.92 (H-19, s), 6.80 (H-21, m), 7.66 (H-22, m), 7.15 (H-2′, brs), 7.53 (H-4′, d, J=7.9 Hz), 7.01 (H-5′, 7.09 (H-6′, t), 7.38 (H-7′, d, J= 8.15 Hz), 2.11 (19-O-acetyl);^{13}C NMR (175 MHz, methanol-d4) δH^{13}C 59.0 (C-3), 48.4 (C-4), 127.1 (C-5), 134.0 (C-6), 69.2 (C-7), 52.3 (C-8), 62.2 (C-9), 33.2 (C-10), 17.7 (C-11), 14.6 (C-12), 129.1 (C-13), 135.5 (C-14), 41.9 (C-15), 33.0 (C-16), 143.3 (C-17), 127.9 (C-18), 84.2 (C-19), 194.9 (C-20), 135.0 (C-21), 136.8 (C-22), 199.7 (C-23), 172.5 (C-24), 124.5 (C-2′), 111.7 (C-3′), 119.2(C-4′), 119.6 (C-5′), 122.3 (C-6′), 112.3 (C-7′), 169.4 (19-O-acetyl C=O), 20.2 (19-O-acetyl –Me), 137.1 (C-1a′), 128.0 (C-3a′).
